# Efficacy of auriculotherapy for decreasing anxiety and stress among perioperative nursing workers: a mixed study*

**DOI:** 10.1590/1518-8345.7218.4275

**Published:** 2024-09-02

**Authors:** Oclaris Lopes Munhoz, Bruna Xavier Morais, Emanuelli Mancio Ferreira da Luz, Patrícia Bitencourt Toscani Greco, Silomar Ilha, Tânia Solange Bosi de Souza Magnago

**Affiliations:** 1 Universidade Federal do Rio Grande, Escola de Enfermagem, Rio Grande, RS, Brazil.; 2 Hospital da Brigada Militar de Santa Maria, Santa Maria, RS, Brazil.; 3 Universidade Federal de Santa Maria, Departamento de Ciências da Saúde, Palmeira das Missões, RS, Brazil.; 4 Universidade Federal de Santa Maria, Santa Maria, RS, Brazil.; 5 Scholarship holder at the Conselho Nacional de Desenvolvimento Científico e Tecnológico (CNPq), Brazil.

**Keywords:** Anxiety, Occupational Stress, Auriculotherapy, Perioperative Nursing, Occupational Health, Nursing

## Abstract

**Objective:**

to analyze the effectiveness of auriculotherapy for decreasing anxiety and stress of perioperative nursing professionals.

**Method:**

mixed methods research, embedded experimental model. In the quantitative stage, a randomized, triple-blind clinical trial was conducted with perioperative nursing professionals, who answered a characterization questionnaire, the List of Signs and Symptoms of Stress*,* and the General Anxiety Disorder-GAD 7. The participants attended eight auriculotherapy sessions with semi-permanent needles. The qualitative stage was exploratory and descriptive, in which data were collected through semi-structured interviews. Data were mixed with the incorporation of qualitative findings to examine the intervention in the experimental study.

**Results:**

13 professionals participated in the intervention group and 14 in the control group. Anxiety and stress levels decreased significantly within groups, though no statistical difference was found between groups (p>0.05). The central category, “Auriculotherapy as an intervention to treat anxiety and stress,” emerged from the qualitative data, which was subdivided into a base unit and three categories concerning the therapy’s benefits.

**Conclusion:**

applying real and sham auriculotherapy had the same effect on the participants’ anxiety and stress levels; the reports reinforced such evidence. Non-pharmacological interventions, such as auriculotherapy, are essential for recovering and promoting the health of perioperative nursing professionals. Brazilian Clinical Trials Registry: RBR-3jvmdn.

## Highlights

**(1)** Significant decrease in intragroup anxiety and stress, with no differences between groups.

**(2)** Real and sham auriculotherapy presented similar effects.

**(3)** Mixed study, incorporating qualitative data to investigate the intervention.

**(4)** Pioneering data on auriculotherapy, anxiety, and stress in perioperative units.

**(5)** Non-pharmacological interventions are essential to promote the health of workers.

## Introduction

The historical evolution of occupational health is permeated by achievements and benefits aimed at better work conditions^1^. However, some environments are still affected by factors that predispose workers to mental illnesses, such as healthcare facilities, where nursing professionals are amongst the most affected^2^ ^)-(^ ^3^.

The work conditions of perioperative care centers predispose nursing professionals to the development of anxiety and stress^3^ ^)-(^ ^4^. Such conditions are possibly related to understaffing, interprofessional conflicts, and the need for specific clinical-care knowledge. Surgical centers, post-anesthesia care units, and sterile and materials processing departments are settings in which nursing workers are more frequently exposed to anxiety and stress^3^ ^),(^ ^5^ ^)-(^ ^6^.

Anxiety is characterized by fear and vague and unpleasant feelings, generally related to anticipated suffering, tension, and discomfort^7^. Stress is related to psychological conditions and demands to which an individual is exposed, compromising one’s psychological health^8^. As a rule, such conditions require specialized therapeutic care, the use of medications and coping strategies^7^ ^)-(^ ^8^, aspects that require adherence, behavioral changes, social conditions, and availability of time, which are challenging aspects when one faces such problems; hence, the importance of alternative interventions.

In this context, auriculotherapy benefits health^9^ ^)-(^ ^13^. It is an ancient technique that uses different spherical or pointed materials to stimulate the ear^9^, which has reflex-somatic connections to the Central Nervous System. Hence, homeostasis is favored^9^ when specific points are stimulated. Furthermore, it is a low-cost practice with minimal/temporary side effects and can be applied in a short period of time^9^ ^)-(^ ^10^.

Research has shown evidence^10^ ^)-(^ ^13^ regarding the use of auriculotherapy, identifying its effectiveness in reducing chronic pain in the spine^13^, alleviating anxiety and stress^10^ ^)-(^ ^11^, and in promoting the quality of life of nursing workers^12^. However, research addressing the practice in perioperative care settings, concomitantly measuring these problems, and using mixed methods is incipient^10^. Thus, the relevance of this study lies in the analytical gain of the multi-method approach, where qualitative secondary data explains the effects of an experiment, producing more robust inferences, and is an innovative feature for future studies.

Considering the gaps in this field^10^, the objective was to analyze the effectiveness of auriculotherapy in treating anxiety and stress of perioperative nursing professionals.

## Method

### Study design

Mixed-methods research using the incorporated experimental strategy QUAN (qual)^14^, in which qualitative data were incorporated to examine the intervention process in the quantitative study^14^. In the quantitative stage, a randomized clinical trial (RCT), triple-blind (patient, statistician, and outcome evaluators) was developed. A complementary qualitative, exploratory, and descriptive stage was also performed.

The CONSORT (Consolidated Standards of Reporting Trials)^15^, COREQ (Consolidated Criteria for Reporting Qualitative Research)^16^, and MMAT (Mixed Methods Appraisal Tool)^17^ guidelines were followed.

### Study setting

This study setting included the Surgical Center (SC), the Post-Anesthesia Care Unit (PACU), the General Surgical Unit (GSU), and the Sterile and Materials Processing Department (SMPD) of a teaching hospital in Rio Grande do Sul.

### Selection and sample criteria

The inclusion criteria adopted for the RCT were: being a nursing worker from one of the units previously mentioned; being available to attend auriculotherapy sessions; experiencing anxiety symptoms (scores ≥ 10 are considered positive for the occurrence of generalized anxiety disorder)^18^; and at least an average stress level (score ≥ 29)^19^. Those with injuries or inflammation in the ear or who started using psychotropic medication or other therapy to treat anxiety and/or stress during the study period or up to 30 days before data collection was initiated were excluded to prevent confounding effects. Seventh-four nursing workers were approached, 15 refused to participate, and two were excluded for having started treatment for anxiety. Thus, 57 were assessed for eligibility, and 27 met the selection criteria.

Eight participants of the experimental stage were included in the qualitative stage through a draw: four from the intervention group (IG) and four from the placebo group (PG).

### Data collection

Quantitative data were collected between October 2022 and March 2023. The professionals were invited to fill out the instruments at their work unit. The outcome evaluators, unaware of the randomization and responsible for assessing whether the participants met the selection criteria, applied the instruments (delivered the forms to the participants so they could fill them out). Once a participant met the criteria, s/he was immediately informed, invited, and included in the intervention. 

The practitioners implementing the auriculotherapy followed this process and had the envelopes (sealed, opaque, and consecutively numbered) containing the group to which each participant would be included. The envelope was opened in the participant’s presence, though the participants remained unaware of the group to which they were included. Randomization was performed by a researcher external to the research, using Random Allocation Software (RAS) in two blocks (of 12 and 16 numbers, respectively).

After randomization, the IG and PG participants were invited to attend individual sessions when the technique would be applied. Each worker attended eight sessions (two per week) lasting between 10 and 15 minutes. The sessions took place during the participants’ work shifts (morning, afternoon, or night; i.e., the participants attended the sessions according to their shifts). The participants were assessed on the 1^st^, 4^th^, and end of the eight sessions, and 15 days after the applications had ceased (follow-up). The number of sessions and the follow-up were established according to a meta-analysis performed earlier^10^. The same collectors applied the outcome assessment questionnaires on these occasions.

The following forms were used to collect quantitative data: a profile instrument (addressing the participants’ age, marital status, job role, intention to quit, weight gain in the last six months, physician exercise, time for leisure, and illness diagnosed by a doctor); the GAD 7 - General Anxiety Disorder, which assesses generalized anxiety considering the last two weeks, according to a seven-point Likert scale: 0 (none), 1 (several days), 2 (more than half of the days), and 3 (almost every day)^18^; and the Signs and Symptoms of Stress List (LSS), 60 items, indicating the frequency with which stress symptoms are perceived: 0 (never), 1 (rarely), 2 (often), and 3 (always)^19^.

Regarding the auriculotherapy protocol applied in each group, four qualified practitioners adequately trained in the technique (two with five years of experience and two with one year) applied the protocols. The points chosen for the intervention and placebo groups were determined according to the literature^10^: the *Shen Men*, brainstem, kidney, sympathetic, and liver points (which have sedative, analgesic, and anxiolytic effects on emotions and balance)^9^ were adopted for the IG. In turn, the cheek, external nose, rectum, ankle, and trachea points (each with a specific indication to intervene on changes related to the anatomical region of its definition) were chosen for the CG^9^.

An electrodiagnostic device, brand EL30 Finder Basic-NKL, was used to identify the points in the IG precisely. In turn, the sites that would elicit the most minor response were chosen for the PG^20^. The procedure included disinfecting the participants’ auricles using anatomical tweezers wrapped in cotton wool soaked in 70% alcohol. Next, semi-permanent needles, brand X Press, size 0.18 x 1.4 mm, were inserted and covered with round, hypoallergenic tape. The participants were instructed about necessary self-care^20^. Four participants (three from IG and one from PG) reported the loss of one auricular needle in one of their sessions; as instructed, they did not try to replace the semi-permanent needle. Additionally, three members of the IG and two of the PG reported intense pain on the first day of application, and in another two different sessions, but chose not to contact the practitioner, as the symptoms subsided within one day after the application; there were three other cases of local pruritus.

Qualitative data were collected between January and March 2023. Those selected (according to previously mentioned criteria) to participate in this stage were invited to respond to the semi-structured interview at their workplaces, and no one refused to participate.

The research team developed and then tested a specific script with two research group members. The script asked: How did you feel before receiving the auriculotherapy intervention? How did you feel, or how are you feeling after the intervention? Have you noticed any changes in your health or daily behavior after the auriculotherapy sessions? Have you experienced any discomfort or symptoms related to auriculotherapy?

A technically experienced researcher with a doctorate in nursing and one undergraduate nursing student held the interviews in a private, noise-free room. The interviews were audio recorded and lasted 23 minutes on average. Microsoft Office Word^®^ was used to transcribe the interviews verbatim. Two authors reviewed the interviews and made some necessary adjustments to the language. Theoretical saturation^21^ was the criterion considered, i.e., data collection ceased when aspects concerning the contributions of auriculotherapy were repeatedly reported.

### Treatment and analysis of data

Two previously trained individuals independently entered the quantitative data into the Excel program and checked for inconsistencies. Next, the Statistical Package for the Social Sciences (SPSS), version 18.0, was used to process data. The variables concerning the participants’ profiles were presented with absolute and relative frequencies or mean and standard deviation.

The scores of GAD-7 and LSS, obtained by summing the items’ points, were analyzed. Scores ≥ 10 indicate generalized anxiety disorder. Anxiety was categorized as minimal (0 to 4 points), low (from 5 to 9), moderate (from 10 to 14), or severe (from 15 to 21 points)^18^. No stress was characterized by a score ranging from 0 to 11; low stress, when scores were from 12 to 28; moderate, from 29 to 60; a score between 61 and 120 concerned a high-stress level; and scores above 120 concerned a very high-stress level^19^.

Thirteen participants from the IG and 14 from the PG were included in the analysis of the anxiety outcome. A previous study^10^ reports that the response within each group was normally distributed with a standard deviation of 5.58. Considering a clinically relevant difference in anxiety between the groups of 7.14, a power of 0.87 was calculated for this study’s sample. The stress response within each group was normally distributed with a standard deviation of 36.36^10^; the power for the sample was 0.36 based on a clinically relevant difference of 24.5. The type I error probability associated with this null hypothesis test is 0.05.

The Chi-square test, Fisher’s exact test, and independent samples t-test were adopted to assess the groups’ homogeneity regarding sociodemographic characteristics, work, health characteristics, lifestyle, and initial anxiety and stress levels.

An intention-to-treat and protocol analysis was implemented to assess the outcome data obtained in the 1^st^, 4^th^, and 8^th^ sessions and follow-up. Multiple imputation was adopted to identify missing data over time. The IG and PG were compared using linear mixed models. A suitable (unstructured) covariance structure was tested. The model parameters were estimated using maximum probability^22^, and statistical significance was set at 0.05 for all tests. A professional external to the research group conducted the analysis.

Discursive Textual Analysis^23^, which includes unitarization, establishment of relationships, and communication, was adopted to analyze the statements. A central category was identified, from which a base unit and three categories of analysis emerged.

Qualitative data were incorporated to complement and deepen the analysis of the experimental study. Diagrams were used as an analytical resource to display the results of the two approaches and the combination of both (joint-display)^24^.

### Ethical aspects

The participants were included only after receiving clarification about the study’s objectives and signing two copies of the informed consent form. The letters “B” (block) and “P” (participant), followed by an alphanumeric number, were used to identify the participants and ensure confidentiality (i.e., codes B1P2, B1P4, B2P2, and B2P3 correspond to the IG and codes B1P1, B1P3, B2P1, and B2P4 to the PG). After data collection and analysis ceased, the PG participants were instructed about their sham condition and were offered the intervention protocol for the same period and number of sessions as the IG. Two members agreed to receive the treatment.

This study was approved by the Institutional Review Board, under opinion No. 3,897,861 and CAAE No. 22328819.8.0000.5346, in March 2020 and registered in the Brazilian Registry of Randomized Clinical Trials (REBEc) under code RBR-3jvmdn.

## Results

**Figure 1:**
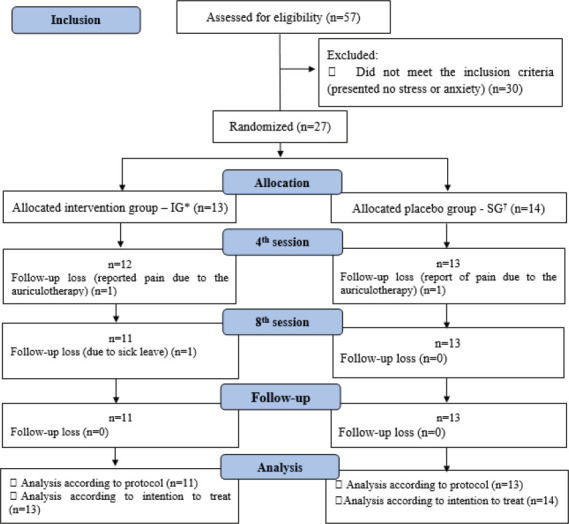
Flowchart concerning the participants’ allocation in the randomized trial, adapted from the Consolidated Standards of Reporting Trials^15^. Santa Maria, RS, Brazil, 2022-2023 *IG = Intervention Group; ^†^PG = Placebo Group

Twenty-seven nursing workers participated in the randomization process, 13 of whom were allocated to the IG and 14 to the PG (Figure 1).

A comparison between the IG and PG revealed that the groups’ characteristics were homogeneous (p>0.05) (Table 1).

**Table 1:** Participants’ characterization and prevalence of anxiety and stress in the intervention and control groups (n = 27). Santa Maria, RS, Brazil, 2022-2023

Variables		Intervention Group (n=13)		Placebo Group (n=14)		p-value
		n	%	n	%	
Marital Status	Married	7	53.9	8	57.2	0.928*
	Single	4	30.8	3	21.4	
	Divorced	2	15.3	3	21.4	
Job position	Nurse	5	38.5	3	21.4	0.645^†^
	Nursing technician/aide	8	61.5	11	78.6	
Intention to quit	Yes	2	15.4	2	14.3	0.546*
	No	8	61.5	10	71.4	
	Maybe	3	23.1	2	14.3	
Weight gain in the last six months	Yes	7	53.8	9	64.3	0.704^†^
	No	6	46.2	5	35.7	
Physical exercise	Yes	6	46.2	3	21.4	0.613*
	No	5	38.5	6	42.9	
	Sometimes	2	15.3	5	35.7	
Have time for leisure	Yes	3	23.0	4	28.6	0.851*
	No	5	38.5	3	21.4	
	Sometimes	5	38.5	7	50.0	
Have a diagnosed health condition	Yes	9	69.2	6	42.8	0.168^‡^
	No	4	30.8	8	57.2	
		Mead	SD^§^	Mead	SD^§^	
Age		41.75	8.92	43.38	6.88	0.611^||^
Prevalence of anxiety before the 1^st^ session		13.50	2.61	14.23	3.14	0.535^||^
Prevalence of stress before the 1^st^ session		77.58	16.64	82.69	32.03	0.626^||^

*Chi-square with correction; ^†^Fisher’s exact test; ^‡^Chi-square; ^§^SD = Standard Deviation; ^||^Independent samples t-test

Intragroup anxiety and stress levels decreased significantly between session intervals and follow-up (p<0.05). No differences were found between the groups (p>0.05) (Table 2). The results of the intention-to-treat analysis are similar to those of the protocol.

**Table 2:** Comparison between the anxiety and stress means obtained by the intervention and placebo groups (linear mixed model analysis, per protocol) over four assessments (n = 24). Santa Maria, RS, Brazil, 2022-2023

Outcome	Group	Session (I)	Session (J)	Difference in means (I-J)	p-value
Anxiety*	Intervention	1^st^	4^th^	4.96	**0.001**
			8^th^	5.50	**0.004**
			Follow-up	5.30	**0.000**
		4^th^	1^st^	-4.96	**0.001**
			8^th^	0.53	0.73
			Follow-up	0.33	0.82
		8^th^	1^st^	-5.50	**0.004**
			4^th^	-0.54	0.73
			Follow-up	-0.19	0.88
		Follow-up	1^st^	-5.30	**0.000**
			4^th^	-0.34	0.82
			8^th^	0.19	0.88
	Placebo	1^st^	4^th^	6.46	**0.000**
			8^th^	5.16	**0.004**
			Follow-up	5.83	**0.000**
		4^th^	1^st^	-6.36	**0.000**
			8^th^	-1.30	0.37
			Follow-up	-0.63	0.66
		8^th^	1^st^	-5.16	**0.004**
			4^th^	1.30	0.37
			Follow-up	0.67	0.59
		Follow-up	1^st^	-5.83	**0.00**
			4^th^	0.63	0.66
			8^th^	-0.67	0.59
Stress^†^	Intervention	1^st^	4^th^	14.72	**0.015**
			8^th^	26.65	**0.002**
			Follow-up	17.50	**0.025**
		4^th^	1^st^	-14.72	**0.015**
			8^th^	11.93	**0.016**
			Follow-up	2.78	0.59
		8^th^	1^st^	-26.65	**0.002**
			4^th^	-11.93	**0.016**
			Follow-up	-9.14	0.09
		Follow-up	1^st^	-17.50	**0.025**
			4^th^	-2.78	0.59
			8^th^	9.15	0.09
	Placebo	1^st^	4^th^	22.58	**0.000**
			8^th^	27.00	**0.001**
			Follow-up	27.45	**0.001**
		4^th^	1^st^	-22.58	**0.000**
			8^th^	4.41	0.30
			Follow-up	4.87	0.31
		8^th^	1^st^	-27.00	**0.001**
			4^th^	-4.41	0.30
			Follow-up	0.45	0.93
		Follow-up	1^st^	-27.45	**0.001**
			4^th^	-4.87	0.32
			8^th^	-0.46	0.93

Note: The letters I and J indicate a specific session (I) in comparison to others (J)

*Measured with the General Anxiety Disorder instrument (GAD 7); ^†^Measured with the List of Signs and Symptoms of Stress (LSS)

Qualitative data enabled a deepened understanding of the problems addressed here and identifying the central category: “Auriculotherapy as an intervention for anxiety and stress,” unitized in the base unit “Benefits of auriculotherapy (intervention and sham points) for anxiety and stress among perioperative nursing workers” from which emerged the analysis categories: “Auriculotherapy to treat anxiety and stress in the intervention group”; “Auriculotherapy for anxiety and stress in the placebo group”; and “auriculotherapy side effects.”

### Auriculotherapy to treat anxiety and stress in the intervention group

Before the auriculotherapy intervention was implemented, the participants presented stress and anxiety symptoms, such as tiredness, exhaustion, dyspnea, insomnia, irritability, lack of or excess appetite:

*[...] Before auriculotherapy, I felt irritated, stressed, and exhausted exactly like that* (B1P2).

*I was much more irritated. [...] in an extreme crisis, anxious, stressed [...] there were times when I couldn’t speak and wanted to cry [...] before the intervention, I’d score a 10 in anxiety, stress, and exhaustion, reaching my limit, I couldn’t switch off [...] I’d eat out of obligation, I have hypoglycemia [...] I’d vomited, my stomach would hurt, hurt. I didn’t sleep or eat at home. The whole time, I experienced bruxism, shallow breathing [...] I was exhausted and hungry, but I couldn’t eat. I was really reaching my limit* (B1P4).

*Stress, quite stressed, especially early in the morning [...] I’d take it out on people at home. I’d snap even at my colleagues. And anxiety, I’d feel anxious, so I’d feel like eating or smoking* (B2P3).

Improvement/relief in stress and anxiety symptoms were reported after the auriculotherapy intervention, with positive repercussions on sleep quality and decreased appetite and tobacco use. The participants mentioned coping strategies to deal with the conditions, such as facing problems while respecting their limits, seeking to establish healthy interpersonal relationships with the team and lifestyle:

*[…] I’ve felt much better after the intervention […] I’m much calmer, more peaceful, despite the work being heavy […] what is happening after the intervention is that the problems here in the service remain, only that not now they no longer affect me, I’m trying to do my best without internalizing problems [...] even my sleep has improved [...]* (B1P2).

*[…] Throughout the intervention, even in the first week, I* already felt very different, much lighter and calmer, and the change was quite noticeable. The injuries started healing, my cervical region improved completely, and my abdomen and lower limbs’ problems are also improving. I started feeling better; I no longer feel anguish, chest tightness, suffocation, that feeling of not being able to speak [...] anxiety; I can say that it has reached zero, I am super calm and peaceful, my mind is quiet, one is not able not feeling stressful at work, but on a scale from 0 to 10, it is between 1 and 2, exhaustion has also decreased, I no longer feel exhausted, just feel naturally tired during the day [...] (B1P4).

*I felt a lot of improvement in my anxiety [...] I’m smoking less frequently, I’ve lost a lot of weight [...] in terms of irritation, even people are saying that I’m calmer, talking less, I’m in a more “Zen state” so to speak, at home too, I’m not stressing out anymore, I’m calm [...] I believe that auriculotherapy had an effect; from my point of view, it helped me [...] I started taking better care of myself. I’m eating better, I’ve started exercising, I’ve started playing soccer again, and I’m less stressed [...] I had a lot of pain in my stomach linked to stress. I didn’t have the persistence to do things before, but after it, it seems like my head was brainwashed, and now, I have a goal, and I’m managing to do it* (B2P3).

One participant could not identify whether the perceived improvement occurred due to the intervention or fewer stressors at work.

*[...] I can’t tell you if it got better or worse [...] I didn’t notice much difference because I also think that we are in a very turbulent period here [referring to the service] [...]* (B2P2).

### Auriculotherapy for anxiety and stress in the placebo group

Before receiving the sham treatment, the participants reported stress and anxiety symptoms, which would manifest physically, psychologically, and behaviorally:

*I was feeling very anxious; I’ve been really anxious for days [...] life is busy, so this creates a lot of stress and anxiety, both at work and in your personal life* (B1P1).

*Before receiving the treatment, I was very stressed and a little unmotivated too [...]. I experienced tachycardia and sweat and had nightmares about work* (B1P3).

*[…] The situations that happen at work end up irritating us. […] I’d feel sad; I think I was a little depressed, eating a lot, and anxious, even now in the last* few days (B2P1).

*I felt pretty nervous, worried about several things, having anxiety attacks, worried about the work overload, and everything I had to do [...] I experienced tremors, headaches, muscle pain, tachycardia, vertigo, and felt a lot like crying […]* (B2P4).

Next, Table 3 presents the intergroup analysis concerning the prevalence of anxiety and stress symptoms experienced throughout the four assessments among the participants who completed the intervention protocol.

**Table 3:** Descriptive of mean and standard deviation of anxiety and stress levels in the four assessments (n = 24). Santa Maria, RS, Brazil, 2022-2023

Outcome	Group	n	1^st^ session	4^th^ session	8^th^ session	Follow-up
			Mean (SD*)	Mean (SD*)	Mean (SD*)	Mean (SD*)
Anxiety	Intervention	11	13.5(2.6)	8.7(4.7)	7.4(4.7)	9.4(4.1)
	Placebo	13	14.2(3.1)	9.0(5.5)	9.0(6.8)	7.6(5.1)
Stress	Intervention	11	77.5(16.6)	63.5(18.5)	51.2(27.9)	62.1(24.4)
	Placebo	13	82.6(32.0)	59.4(31.5)	54.9(32.2)	50.3(26.1)

*SD = Standard Deviation

The reports show that some participants did not identify a relief/decrease in symptoms at the end of the auriculotherapy sessions addressing sham points. However, some did report improved willingness to work after the sham sessions:

*[…] I don’t know if it was a placebo or an intervention, but so far I’m a little calmer, trying to do things more calmly, not rushing through everything, because there’s no point […] I even joked that my [auriculotherapy treatment] was a placebo, because I was really very anxious [...] but now I’m better* (B1P1).

*[...] I’ve noticed a very satisfactory improvement during the intervention, because I became more willing to work, calmer, less anxious […] I believe that when I was in the treatment period I felt more willing at work and for everything, now it seems that something is missing* (B1P3).

*Look, what I’m going to tell you, I couldn’t notice [...] but my colleagues did* (B2P1).

*[…] I felt better, but I don’t know if it had to do with the auriculotherapy or with decreased responsibilities […] I didn’t notice a change in my health behavior after the auriculotherapy sessions* (B2P4).

### Auriculotherapy side effects

The side effects the IG and PG reported included local symptoms that are usually expected when this technique is applied, such as discomfort, pain, and/or itching on the site due to direct contact or specific points.

*I experienced discomfort when the needles were inserted. Locally, it’s not pain, nothing, just discomfort, nothing much* (B1P1).

*[…] Some points were sore to the touch, especially those on the inside of the ear, but they were painful to the touch. I mean, I felt no pain; it wouldn’t hurt unless I touched it* (B1P2).

*[…] I experienced a lot of discomfort at times […] it was bothering me a lot, a lot of pain, the points would itch […] sometimes, but not always* (B1P3).

*There were sore points, but a more specific point was painful, and it improved with the sessions. It wasn’t unbearable […] nothing out of the ordinary […] I didn’t do anything* [to relieve the pain]; *I just waited* (B1P4).

*[…] The only discomfort I experienced was in my left ear, which was more sensitive, so I preferred applications on the right ear […] it was more bearable* (B2P1).

*[…] When you forget and touch it with your hand […], sometimes it gets more sore, some of the points, particularly those on the inside of the ear, but nothing major* (B2P2).

Figure 2 presents the meta-inferences derived from the reports regarding the quantitative results. 

**Figure 2:** Joint display representing the combination of data and meta-inferences

QUAN* Results	Qual^†^ Results	Meta-inferences
Auriculotherapy significantly decreased intragroup anxiety and stress symptoms between the 1^st^ and 8^th^ sessions and the follow-up (Table 1).	Base unit: “Benefits of auriculotherapy (intervention and placebo points) for anxiety and stress among perioperative nursing workers.”	Perioperative nursing workers reported significant stress and anxiety symptoms before the intervention. Auriculotherapy showed positive results in decreasing intragroup stress and anxiety, i.e., both for the intervention and placebo groups, reinforcing the benefits of non-pharmacological practices.
A difference in anxiety levels was found in the Intervention Group between the 1^st^, 4^th^ (p^‡^=0.001), 8^th^ sessions (p^‡^=0.004) and the follow-up (p^‡^=0.001); between the 4^th^ and 1^st^ (p^‡^=0.001); between the 8^th^ and 1^st^ sessions (p^‡^=0.004); and, between follow-up and 1^st^ session (p^‡^=0.000). Differences regarding stress were found between the 1^st^, 4^th^ (p^‡^=0.015), 8^th^ sessions (p=0.002) and follow-up (p^‡^=0.025); between the 4^th^ and 1^st^ (p^‡^=0.015) and 8^th^ session (p^‡^=0.016); between the 8^th^ and 1^st^ (p^‡^=0.002) and the 4^th^ sessions (p^‡^=0.016); and, between follow-up and 1^st^ session (p^‡^=0.025) (Table 2).	Category of analysis: “Auriculotherapy to treat anxiety and stress in the intervention group.”	Auriculotherapy was beneficial in controlling anxiety and stress symptoms; no differences were found between the groups, though. It contributed to improving the foremost common symptoms such as sleep disorders, appetite, irritability, and tobacco consumption, improving the participants’ self-perception.
		The participants reported that they started adopting coping strategies to deal with stressors after the auriculotherapy sessions.
Anxiety levels decreased by 44.5% between the 1^st^ and 8^th^ sessions in the Intervention Group, with a slight increase in the follow-up. The Placebo Group showed a decrease of 36.75% in the same period, which remained at the follow-up.	Category of analysis: “Auriculotherapy for anxiety and stress in the placebo group.”	Real and sham point (placebo) stimulation in auriculotherapy produced physiological or belief-related effects for the nursing professionals in the placebo group, as they reported decreased anxiety and stress symptoms. This result is explained by the fact that these conditions tend to respond positively to listening and attention given to those affected. Furthermore, the ankle point was one of the points used in the sham treatment. According to Traditional Chinese Medicine, despite being a specific point used to treat muscle pain in this site, it has an analgesic effect.
Regarding the stress outcome in the Intervention Group, a 34.01% decrease was obtained between the 1^st^ and 8^th^ sessions, though it did not remain at the follow-up. Regarding the Placebo Group, a decrease of 33.67% was found between the 1^st^ and 8^th^ sessions, which remained at the follow-up (Table 3).		Having anxiety or stress symptoms is a unique experience, and how the participants perceive the effects of auriculotherapy is subjective. Some participants reported improved sleep, appetite, and other symptoms, while others did not. Furthermore, this practice improved the individuals’ overall well-being, providing emotional balance, decreasing irritability, and a better disposition for work.
	Category of analysis: “Auriculotherapy side effects.”	Auriculotherapy was minimally invasive, safe, and demanded little time.
		The adverse events linked to the auriculotherapy were minimal. The participants reported temporary, tolerable symptoms, discomfort at the site where the semi-permanent needles were inserted, skin irritation, and hyperemia (redness).

*QUAN = Quantitative; ^†^Qual = Qualitative. ^‡^p = p-value

## Discussion

Both groups reported the beneficial effects of auriculotherapy on decreasing anxiety and stress levels among perioperative nursing professionals. Applying auriculotherapy using real or sham points had the same effects on the anxiety and stress experienced by the study participants. Indices improved over the eight auriculotherapy sessions and follow-up.

The literature on mixed methods research simultaneously addressing the effectiveness of auriculotherapy on anxiety and stress outcomes among perioperative nursing professionals is incipient, hindering comparisons. On the other hand, the few studies in the field highlight that this study is pioneering, revealing the potential of incorporating qualitative data to explain the effects of experimental research and contributing to existing literature.

A study conducted in the United States of America showed that anxiety levels (p<0.05) decreased among health workers who were treated with auriculotherapy in six sessions^11^. In Brazil, Primary Health Care workers also experienced reduced stress levels after receiving the intervention^25^. An investigation addressing nursing professionals working in a hospital setting found decreased anxiety, stress, and depression levels during the COVID-19 pandemic^26^.

These effects are due to the auriculotherapy mechanism of action, which occurs through the stimulation of auricular areas associated with the reticular formation from the sympathetic and parasympathetic nervous systems. Such information is transmitted from the ear by means of stimulation through the fibers responsible for innervations. The vagus nerve is among the primary nerves that send information to brain regions essential for regulating anxiety^27^. 

Regarding evidence that auriculotherapy applied to real and sham points had the same effect on anxiety and stress levels among the professionals addressed here, other studies also found positive effects with the use of sham treatment^10^ ^),(^ ^25^ ^),(^ ^28^. Sham points were also beneficial when used for chronic spinal pain^13^ and depression^28^. On the other hand, there is evidence that specific intervention points for certain conditions elicit better effects than sham auriculotherapy^10^ ^),(^ ^25^.

From this perspective, stimulating any auricular point might trigger physiological effects on individuals’ bodies and produce belief-related effects. Such a result may be explained by neurological mechanisms in which neurotransmitters are released (e.g., opioids, dopamine, and serotonin), which adjust the patients’ biological responses, besides psychological mechanisms, in which emotional conditions, such as anxiety and self-control, changes the individuals’ perception of their health condition, promoting resilience^9^ ^),(^ ^13^ ^),(^ ^27^.

It is worth discussing the points used in the groups and their effects according to Traditional Chinese Medicine^9^. Those in IG received treatment to the *Shen Men*, brainstem, kidney, sympathetic, and liver points, which are shown to be the most frequently used for anxiety, stress, and burnout^10^. The *Shen Men* point has a tranquilizing and analgesic effect, used in the excitatory and inhibitory action of the cerebral cortex; the brain stem stimulates the mind, calms the spirit, helps with brain disorders, and is sedative; the kidney strengthens essential energy and promotes health conservation; the sympathetic system helps with circulatory and neurovegetative changes; and finally, the liver point regulates emotions such as irritability, controls Qi (energy), and activates blood circulation^9^. 

In turn, the points treated in the PG participants were the cheek, external nose, rectum, ankle, and trachea, which are aimed at treating facial disorders; conditions in the nose area, such as inflammations and macules, among others; internal and external hemorrhoids and rectal prolapse; sprains and joint inflammation; and alleviating coughs, eliminating phlegm, and draining the throat, respectively^9^. In other words, these points treat conditions not directly related to anxiety or stress.

In this sense, in parallel with French auriculotherapy, the theory of embryological zones is addressed, in which the ear is projected into three membranes: ectoderm, mesoderm, and endoderm. Associating the points used in this study with this theory, the IG presented two points linked to the mesodermal area, which is innervated by the minor trigeminal occipital and great auricular nerves and are connected to areas of muscle and subcutaneous pain; also, to the endodermal area, innervated by the glossopharyngeal, facial, vagus, and trigeminal nerves, used in the treatment of deep pain^9^.

The relationship between stress and pain outcomes is acknowledged. A study in the healthcare context notes that the stress experienced by nursing workers might permeate psychological aspects and have a physical influence, such as pain. Therefore, pain might be considered a sign of stress associated with anxiety and muscle tension^29^. Hence, considering the points treated in the PG, the participants had areas related to pain stimulated, such as the ankle point, which may have contributed to improving stress and anxiety parameters among these professionals.

Nurses and nursing students who had completed a training course in auriculotherapy (though with a difference in their experience) implemented the intervention. From this perspective, a clinical study with Portuguese students found that the auriculotherapy practitioner’s experience and clinical practice are essential for assessing a clinical outcome^30^. For this reason, an intervention manual was developed to decrease bias and qualify the training of those implementing the technique proposed here^20^.

In addition to the effects on anxiety and stress, auriculotherapy contributed to improving sleep, controlling appetite, decreasing irritability and tobacco use, and improving the participants’ self-perception. Furthermore, after the auriculotherapy sessions, the professionals began using coping strategies to deal with work-related adversities. Note that this practice aims to reestablish the individuals’ energy balance, which benefits health^31^.

Additionally, there were minimal, short-lasting, local side effects, if any. Clinical research confirms this finding, indicating that adverse events related to auriculotherapy are absent or uncommon^28^, showing it is a safe practice. Hence, when side effects occur, they are transient and tolerable, manifesting as discomfort, mild pain, skin irritation, or itching^10^ at the application site^28^.

The previous discussion reveals the importance of using non-pharmacological practices to improve emotional problems, such as anxiety and stress. Auriculotherapy has proven to be a simple, well-accepted practice with positive and beneficial effects on workers’ health. This study’s results show that perioperative nursing workers were stressed and anxious, and these problems were associated with the tasks and activities inherent to their workplaces. Therefore, prioritizing health promotion is essential to improving workers’ mental health, as healthy workers favor safe and qualified healthcare. 

This study complied with the guidelines for experimental and qualitative studies. However, despite the practitioners being trained and having a guiding manual, different therapists administered the auriculotherapy sessions, and in some cases, the needles fell off. Additionally, the statistical power of the stress outcome is low. Such factors constitute limitations; hence, the evidence reported here must be interpreted cautiously.

The benefits of auriculotherapy among perioperative nursing workers include promoting health and decreasing stress and anxiety levels, which contribute to the health and nursing fields. Auriculotherapy was a humane and integral practice with benefits beyond the primary outcomes. Thus, this study’s evidence can support other contexts where auriculotherapy can be implemented to promote workers’ health.

## Conclusion

This study’s results showed the benefits of auriculotherapy for relieving anxiety and stress among perioperative nursing professionals. Real and sham auriculotherapy showed the same effect on the participants’ anxiety and stress levels. Evidence was reinforced by the participants’ reports, which showed the practice’s contribution. The importance of non-pharmacological interventions, such as auriculotherapy, was highlighted, supporting the recovery and promoting these workers’ health.

Future studies are suggested to investigate the effectiveness of auriculotherapy in other settings and among other professions. Another possibility for research is to compare the technique using different materials, such as a semi-permanent needle *versus* seeds or crystals.

## References

[B1] Silva FFV (2021). Comprehensive worker’s health care: limitations, advances, and challenges. Rev Bras Saude Ocup.

[B2] Saragih ID, Tonapa SI, Saragih IS, Advani S, Batubara SO, Suarilah I (2021). Global prevalence of mental health problems among healthcare Workers during the Covid-19 pandemic: A systematic review and meta-analysis. Int J Nurs Stud.

[B3] Munhoz OL, Arrial TS, Barlem EL, Dalmolin GL, Andolhe R, Magnago TS (2020). Occupational stress and burnout in health professionals of perioperative units. Acta Paul Enferm.

[B4] Appel AP, Carvalho ARS, Santos RP (2021). Prevalência e fatores associados à ansiedade, depressão e estresse numa equipe de enfermagem COVID-19. Rev Gaúcha Enferm.

[B5] Silva TL, Gomes JRAA, Corgozinho MM (2021). Level of stress in nursing professionals of a surgical center. Rev SOBECC.

[B6] Ghawadra SF, Adbullan KL, Choo WY, Phang CK (2019). Psychological distress and its association with job satisfaction among nurses in a teaching hospital. J Clin Nurs.

[B7] Frota IJ, Fé AACM, Paula FTM, Moura VEGS, Campos EM (2022). Anxiety disorders: history, clinical features, and current classifications. J Health Biol Sci.

[B8] Lu S, Wei F, Li G (2021). The evolution of the concept of stress and the framework of the stress system. Cell Stress.

[B9] Neves ML (2019). Acupuntura auricular e neuromodulação.

[B10] Munhoz OL, Morais BX, Santos WM, Paula CC, Magnago TSBS (2022). Effectiveness of auriculotherapy for anxiety, stress or burnout in health professionals: a network meta-analysis. Rev. Latino-Am. Enfermagem.

[B11] Olshan-Perlmutter M, Carter K, Marx J (2019). Auricular acupressure reduces anxiety and burnout in behavioral healthcare. Appl Nurs Res.

[B12] Moura CC, Lourenço BG, Alves BO, Assis BB, Toledo LV, Ruela LO (2023). Quality of life and satisfaction of students with auriculotherapy in the covid-19 pandemic: a quasi-experimental study. Rev Bras Enferm.

[B13] Morais BX, Munhoz OL, Moreira CHC, Kurebayashi LFS, Lopes LFD, Magnago TSBS (2023). Auriculotherapy for reducing chronic spinal pain in health workers: a clinical trial. Rev. Latino-Am. Enfermagem.

[B14] Creswell JW, Clark VLP (2013). Pesquisa de métodos mistos.

[B15] Schulz KF, Altman DG, Moher D, CONSORT Group (2010). CONSORT 2010 Statement: updated guidelines for reporting parallel group randomised trials. BMC Medicine.

[B16] Souza VR, Marziale MH, Silva GT, Nascimento PL (2021). Translation and validation into Brazilian Portuguese and assessment of the COREQ checklist. Acta Paul Enferm.

[B17] Hong QN, Fàbregues S, Bartlett G, Boardman F, Cargo M, Dagenais P (2018). The Mixed Methods Appraisal Tool (MMAT) version 2018 for information professionals and researchers. Educ Inform.

[B18] Robert L, Spitzer RL, Kurt K, Janet BW, Williams BL (2006). A Brief Measure for Assessing Generalized Anxiety Disorder The GAD-7. Arch Intern Med.

[B19] Ferreira EAG, Vasconcellos EG, Marques AP (2002). Assessment of pain and stress in fibromyalgia patients. Rev Bras Reumatol.

[B20] Munhoz OL, Morais BX, Uminski JC, Ilha S, Magnago TSBS (2023). Use of auriculotherapy intervention manual in a randomized clinical trial: experience report. Rev Cient Enferm.

[B21] Fontanella BJB, Luchesi BM, Saide MGB, Ricas J, Turato ER, Melo DG (2011). Sampling in qualitative research: a proposal for procedures to detect theoretical saturation. Cad Saúde Pública.

[B22] Londero AB, Reiniger AP, Tavares RC, Ferreira CM, Wikesjo UME, Kantorski KZ (2022). Efficacy of dental floss in the management of gingival health: a randomized controlled clinical trial. Clin Oral Investig.

[B23] Moraes R, Galiazi MC (2020). Análise textual discursiva.

[B24] Oliveira JLC, Magalhães AMM, Mastuda LM, Santos JLG, Souta RQ, Riboldi CO (2021). Mixed Methods Appraisal Tool: strengthening the methodological rigor of mixed methods research studies in nursing. Texto Contexto Enferm.

[B25] Damasceno KSM, Oliveira GM, Beltrame M, Coelho JMF, Pimentel RFW, Merces MC (2022). Effectiveness of auriculotherapy on stress reduction in health workers: a controlled randomized clinical trial. Rev. Latino-Am. Enfermagem.

[B26] Oliveira CMC, Assis BB, Mendes PG, Lemos IC, Sousa ALC, Chianca TCM (2021). Auriculotherapy in nursing professionals during the coronavirus pandemic: a multiple case study. Rev Eletr Enferm.

[B27] Vieira A, Moreira A, Machado JP, Robinson N, Hu XY (2022). Is auriculotherapy effective and safe for the treatment of anxiety disorders? - A systematic review and meta-analysis. Eur J Integr Med.

[B28] Rodrigues DMO, Menezes PR, Silotto AEMR, Heps A, Sanches NMP, Schveitzer MC (2023). Efficacy and Safety of Auricular Acupuncture for Depression: A Randomized Clinical Trial. JAMA Netw Open.

[B29] Rhoden DJ, Colet CF, Stumm EMF (2021). Association and correlation between stress, musculoskeletal pain and resilience in nurses before hospital accreditation maintenance assessment. Rev. Latino-Am. Enfermagem.

[B30] Vieira A, Sousa P, Moura A, Lopes L, Silva C, Robinson N (2022). The Effect of Auriculotherapy on Situational Anxiety Trigged by Examinations: A Randomized Pilot Trial. Healthcare.

[B31] Bassi MVM, Boaretto JP, Martins EAP (2023). Effectiveness of auriculotherapy in the care of anxiety and stress in nursing professionals: an integrative review. Peer Review.

