# Supplementary material for: Efficacy of auriculotherapy for decreasing anxiety and stress among perioperative nursing workers: a mixed study
Source: Rev Lat Am Enfermagem. 2024 Sep 2;32:e4275. doi: 10.1590/1518-8345.7218.4275 (PMC11386487; doi:10.1590/1518-8345.7218.4275)
Supplement: Translation

# Translation: Eficácia da auriculoterapia para ansiedade e estresse em profissionais de enfermagem perioperatória: estudo misto*

**DOI:** 10.1590/1518-8345.7218.4276

**Authors:** Oclaris Lopes Munhoz, Bruna Xavier Morais, Emanuelli Mancio Ferreira da Luz, Patrícia Bitencourt Toscani Greco, Silomar Ilha, Tânia Solange Bosi de Souza Magnago

**Affiliations:** 1 Universidade Federal do Rio Grande, Escola de Enfermagem, Rio Grande, RS, Brasil.; 2 Hospital da Brigada Militar de Santa Maria, Santa Maria, RS, Brasil.; 3 Universidade Federal de Santa Maria, Departamento de Ciências da Saúde, Palmeira das Missões, RS, Brasil.; 4 Universidade Federal de Santa Maria, Santa Maria, RS, Brasil.; 5 Bolsista do Conselho Nacional de Desenvolvimento Científico e Tecnológico (CNPq), Brasil.

**Keywords:** Ansiedade, Estresse Ocupacional, Auriculoterapia, Enfermagem Perioperatória, Saúde Ocupacional, Enfermagem

## Abstract

**Objetivo:**

analisar a eficácia da auriculoterapia na ansiedade e estresse de profissionais de enfermagem perioperatória.

**Método:**

pesquisa de métodos mistos, experimental incorporada. Na etapa quantitativa realizou-se um ensaio clínico randomizado, triplo cego, com profissionais de enfermagem perioperatória, que responderam a um questionário de caracterização, a Lista de Sinais e Sintomas de Estresse, o Transtorno de Ansiedade Geral-GAD 7, sendo submetidos a oito sessões de auriculoterapia com semiagulhas. A etapa qualitativa foi exploratória e descritiva, com dados obtidos por entrevistas semiestruturadas. Dados mixados com incorporação dos achados qualitativos para examinar a intervenção no estudo experimental.

**Resultados:**

participaram 13 profissionais no grupo intervenção e 14 no controle, constatando-se redução significativa de ansiedade e estresse intragrupos, porém, sem diferença estatística intergrupos (p>0,05). Dados qualitativos possibilitaram a construção da categoria central “Auriculoterapia como intervenção para ansiedade e estresse”, subdividida em uma unidade de base e três categorias, que versaram sobre os benefícios da prática.

**Conclusão:**

aplicar auriculoterapia com pontos verdadeiros e falsos (*sham*) apresentou igual efeito para ansiedade e estresse dos participantes investigados; evidências reforçadas pelos depoimentos. Intervenções não farmacológicas, como a auriculoterapia, são importantes para recuperação e promoção da saúde de profissionais de enfermagem perioperatória. Registro Brasileiro de Ensaios Clínicos: RBR-3jvmdn.

## Destaques

**(1)** Redução significativa de ansiedade e estresse intragrupos, sem diferença entre eles.

**(2)** Auriculoterapia com pontos verdadeiros e falsos (sham) apresentou igual efeito.

**(3)** Estudo misto, com dados qualitativos incorporados, para examinar uma intervenção.

**(4)** Dados pioneiros de auriculoterapia, ansiedade e estresse em unidades perioperatórias.

**(5)** Intervenções não farmacológicas são importantes na promoção da saúde de trabalhadores.

## Introdução

A evolução histórica da saúde do trabalhador é permeada por conquistas e benefícios direcionados a melhores condições nos processos laborais^1^. Por outro lado, há ambientes em que ainda permeiam fatores que predispõem os trabalhadores ao adoecimento psíquico, como o caso de instituições de assistência em saúde, estando os profissionais de enfermagem entre os mais acometidos^2^ ^)-(^ ^3^.

As unidades em que ocorre o cuidado perioperatório apresentam condições que predispõem ao desenvolvimento de ansiedade e estresse em profissionais de enfermagem^3^ ^)-(^ ^4^. Estes agravos podem relacionar-se ao número limitado de equipe, aos conflitos interprofissionais, e à necessidade de conhecimentos clinico-assistenciais específicos. Blocos cirúrgicos, salas de recuperação anestésica e centros de materiais e esterilização representam setores nos quais a enfermagem está mais exposta à ansiedade e ao estresse^3^ ^),(^ ^5^ ^)-(^ ^6^.

A ansiedade caracteriza-se por medo, sentimentos vagos e desagradáveis, geralmente relacionados a sofrimentos antecipados, a tensões e desconfortos^7^. O estresse relaciona-se a condições e demandas psicológicas a que um indivíduo é exposto, comprometendo sua saúde psíquica^8^. Por norma, tais condições necessitam de cuidado terapêutico especializado, uso de medicações e estratégias de enfrentamento^7^ ^)-(^ ^8^, aspectos que requerem adesão, mudanças de atitudes, condições sociais e tempo disponível, características desafiadoras ao enfrentamento destes agravos. Logo, verifica-se a importância de intervenções alternativas.

Neste contexto, a auriculoterapia é uma prática com benefícios à saúde^9^ ^)-(^ ^13^. Trata-se de uma técnica milenar que utiliza diferentes materiais esféricos ou pontiagudos para estimular a orelha^9^, que possui conexões reflexo-somáticas vinculadas ao Sistema Nervoso Central; uma vez estimulados pontos específicos, a homeostase é favorecida^9^. Ainda, é uma prática pouco onerosa, com efeitos colaterais mínimos/passageiros, sendo aplicada em pouco tempo^9^ ^)-(^ ^10^.

Pesquisas têm revelado evidências^10^ ^)-(^ ^13^ acerca do uso da auriculoterapia, identificando eficácia para redução de dor crônica na coluna vertebral^13^ e de ansiedade e estresse^10^ ^)-(^ ^11^, e na promoção da qualidade de vida de profissionais de enfermagem^12^. No entanto, investigações sobre a prática nos cenários de cuidado perioperatório, com mensuração concomitante destes agravos e com métodos mistos, são incipientes^10^. Assim, a relevância desta pesquisa se dá no ganho analítico da abordagem multimétodo, onde os dados secundários qualitativos explicam os efeitos de um experimento, produzindo inferências mais robustas, característica inovadora para estudos futuros. 

Considerando as lacunas da área já mencionadas^10^, objetivou-se analisar a eficácia da auriculoterapia na ansiedade e estresse de profissionais de enfermagem perioperatória.

## Método

### Delineamento do estudo

Pesquisa de métodos mistos, com uso da estratégia experimental incorporada QUAN (qual)^14^, na qual os dados qualitativos foram incorporados para examinar o processo da intervenção no estudo quantitativo^14^. Um ensaio clínico randomizado (ECR), triplo cego (paciente, estatístico e avaliadores de desfecho), foi desenvolvido na etapa quantitativa; uma etapa qualitativa, exploratória e descritiva teve papel complementar.

Foram seguidas as diretrizes CONSORT (*Consolidated Standards of Reporting Trials*)^15^, COREQ (*Consolidated Criteria for Reporting Qualitative Research*)^16^ e MMAT (*Mixed Methods Appraisal Tool*)^17^.

### Cenário do estudo

Constituíram-se cenários o Bloco Cirúrgico (BC), a Sala de Recuperação Anestésica (SRA), a Unidade de Cirurgia Geral (UCG) e a Unidade de Processamento de Materiais e Esterilização (UPME), de um hospital de ensino do Rio Grande do Sul.

### Critérios de seleção e amostra

Para o ECR, constituíram-se critérios de inclusão: ser profissional de enfermagem das unidades supracitadas; possuir disponibilidade para a realização das sessões de auriculoterapia; ter ansiedade (valores ≥ 10 são considerados positivos para a ocorrência do transtorno de ansiedade generalizada)^18^ e no mínimo nível médio de estresse (valores ≥ 29 pontos)^19^. Excluíram-se participantes com lesões ou inflamações no pavilhão auricular e/ou que começaram o uso de medicamento psicotrópico ou outra terapia para o tratamento de ansiedade e/ou estresse no período do estudo ou em até 30 dias antes do início da coleta de dados (visando evitar confundimento de efeitos). Foram acessados 74 profissionais de enfermagem: 15 recusaram-se a participar e dois foram excluídos por iniciarem tratamento para ansiedade. Assim, 57 foram avaliados para elegibilidade e 27 preencheram os critérios de seleção. 

Na etapa qualitativa, incluíram-se oito profissionais participantes da etapa experimental, por meio de sorteio: quatro do grupo intervenção (GI) e quatro do placebo (GP).

### Coleta de dados

Os dados quantitativos foram coletados entre outubro de 2022 e março de 2023. Os profissionais foram convidados a preencher os instrumentos no próprio trabalho/unidade. Aplicaram-se os instrumentos de pesquisa (entregues aos participantes para que pudessem preenchê-los) por avaliadores de desfecho (coletadores), que não possuíam conhecimento sobre a randomização, bem como ficaram responsáveis por avaliar se um determinado participante preencheu os critérios de seleção. Quando os participantes atendiam aos critérios estabelecidos, eram imediatamente informados, convidados e incluídos para a intervenção.

Os intervencionistas de auriculoterapia acompanhavam esse processo e estavam de posse dos envelopes (lacrados, opacos e numerados, consecutivamente), contendo o grupo ao qual o participante incluído faria parte. O envelope era aberto na frente do participante, sem que ele tivesse conhecimento sobre o grupo a ser incluído. A randomização foi desenvolvida em dois blocos (de 12 e 16 números, respectivamente), por pesquisador externo à pesquisa, com o *Random Allocation Software* (RAS).

Após a randomização, os participantes do GI e do GP foram convidados a participar das sessões de aplicação da técnica, de forma individualizada. Cada profissional recebeu oito sessões, duas por semana, com duração entre 10 e 15 minutos, em seus respectivos turnos de trabalho (manhã, tarde ou noite, sendo os turnos mantidos nas aplicações). Os participantes foram avaliados na 1ª, 4ª e ao final das 8 sessões, e 15 dias após o término das aplicações (*follow-up*); número de sessões e acompanhamento foram estabelecidos conforme metanálise previamente realizada^10^. Os questionários de avaliação de desfecho foram aplicados nesses momentos pelos mesmos coletadores. 

Utilizaram-se os seguintes questionários para coleta de dados quantitativos: instrumento de perfil (idade, estado civil, cargo, intenção de deixar o emprego, ganho de peso nos últimos seis meses, prática de atividade física, tempo para lazer e doença diagnosticada por médico); o GAD 7 - Transtorno Geral de Ansiedade, que avalia ansiedade generalizada considerando as últimas duas semanas e possui sete itens em escala Likert, com pontuações de 0 (nenhuma vez), 1 (vários dias), 2 (mais da metade dos dias) e 3 (quase todos os dias)^18^; e, a Lista de Sinais e Sintomas de Estresse (LSS), 60 itens, assinalando-se a frequência com que se percebe sintomas de estresse, com opções de: 0 (nunca), 1 (raramente), 2 (frequentemente) e 3 (sempre)^19^.

Quanto ao protocolo de aplicação da auriculoterapia por grupo, as aplicações foram realizadas por quatro terapeutas capacitados e com formação para a técnica (dois possuíam cinco anos de experiência; dois, um ano). Os pontos de escolha para os grupos intervenção e placebo foram definidos com base na literatura^10^: para o GI, *shen men*, tronco cerebral, rim, simpático e fígado (com efeitos sedativos, analgésicos, ansiolíticos, sobre emoções e equilíbrio)^9^; para o GP, aplicaram-se os pontos bochecha, nariz externo, reto, tornozelo e traqueia (cada um com indicação específica para intervir sobre alterações relacionadas à região anatômica de sua definição)^9^.

Para a detecção dos pontos utilizou-se um aparelho de eletrodiagnóstico, marca *EL30 Finder Basic-NKL*, objetivando melhor precisão dos pontos do GI. Para o GP, foi procurada a área com menor resposta ao estímulo^20^. Ainda, procedia-se com a antissepsia do pavilhão auricular, com pinça anatômica envolta com algodão embebido em álcool 70%. Após, aplicavam-se as agulhas semipermanentes, marca *X Press*, tamanho 0,18 x 1,4 mm, posteriormente cobertas por esparadrapo redondo, hipoalergênico. Os participantes foram orientados acerca dos cuidados necessários com a prática^20^. Quatro participantes (três do GI e um do GP) informaram a perda de um ponto auricular em uma de suas sessões recebidas, e que não tentaram recolocar a semi-agulha, conforme orientado. Também, três integrantes do GI e dois do GP relataram dor intensa no primeiro dia de aplicação, em duas sessões distintas, mas optaram por não contatar o intervencionista, pois o sintoma se reduziu entre o final do dia de aplicação e o posterior. Ocorreram outros três casos de prurido local.

Os dados qualitativos foram coletados entre janeiro e março de 2023. Após o estabelecimento dos participantes para esta coleta (conforme critérios já mencionados anteriormente), procedeu-se com um convite aos profissionais para que respondessem à entrevista semi-estruturada em seus locais de trabalho. Não houve recusas nessa participação.

Um roteiro específico foi desenvolvido pela equipe e testado com dois integrantes de grupo de pesquisa. Incluíram-se os questionamentos: como você se sentia antes de receber a intervenção com auriculoterapia? Como você se sentiu ou se sente após a intervenção? Você notou alguma mudança em sua saúde ou comportamento cotidiano após as sessões de auriculoterapia? Você teve algum desconforto ou algo neste sentido relacionado à auriculoterapia?

Um pesquisador doutor em enfermagem, com experiência na técnica, e uma discente de graduação em enfermagem conduziram as entrevistas em salas reservadas e livres de ruídos. Os depoimentos foram audiogravados e tiveram duração média de 23 minutos. Para as transcrições na íntegra, utilizou-se o *Microsoft Office Word* ^*®*^ . Dois integrantes do estudo revisaram os depoimentos, sendo realizadas adequações necessárias de linguagem. A saturação teórica^21^ foi considerada quando se identificou repetitividade dos aspectos relacionados às contribuições da auriculoterapia.

### Tratamento e análise dos dados

Os dados quantitativos foram digitados no programa *Excel*, de maneira duplo-independente, por pessoal previamente capacitado, com posterior checagem das inconsistências. Após, foram processados pelo *Statistical Package for the Social Sciences* (SPSS), versão 18.0. As variáveis referentes ao perfil da equipe foram apresentadas com frequências absolutas e relativas ou média e desvio-padrão.

O GAD-7 e a LSS foram analisados com a soma de pontos das questões. Para ansiedade, valores ≥ 10 indicam transtorno de ansiedade generalizada presente; ainda, pode-se categorizar em ansiedade mínima (0 a 4 pontos); baixa (de 5 a 9); moderada (de 10 a 14) e severa (de 15 a 21 pontos)^18^. O estresse foi classificado em ausente (de 0 a 11 pontos), baixo (de 12 a 28), médio (29 a 60), alto (61 a 120) e altíssimo nível (acima de 120 pontos)^19^.

Para a análise do desfecho ansiedade, considerou-se a inclusão de 13 participantes no grupo intervenção e 14 no placebo. Em um estudo anterior^10^, a resposta dentro de cada grupo foi normalmente distribuída com desvio-padrão 5,58. Considerando uma diferença clinicamente relevante de ansiedade entre os grupos de 7,14, um poder de 0,87 foi calculado para a amostra deste estudo. Para estresse, a resposta dentro de cada grupo foi normalmente distribuída com desvio-padrão 36,36^10^; tendo por base uma diferença clinicamente relevante de 24,5, o poder calculado para a amostra foi de 0,36. A probabilidade de erro Tipo I associada a este teste desta hipótese nula é de 0,05.

Para avaliação de homogeneidade dos grupos (características sociodemográficas, laborais, de saúde, hábitos de vida e níveis iniciais de ansiedade e estresse), utilizaram-se os testes Qui-quadrado, Exato de Fisher e Teste t de amostras independentes.

No que se refere aos dados de avaliação de desfecho, correspondentes às: 1ª, 4ª e 8ª sessões, e *follow-up*, procedeu-se com análise de intenção de tratar e de protocolo. Utilizou-se de imputação múltipla para estabelecer dados ausentes no tempo. A comparação entre GI e GP foi por meio de modelos lineares mistos. Uma estrutura adequada de covariância (não estruturada) foi testada. Os parâmetros do modelo foram estimados pela probabilidade máxima^22^. O nível de significância estatística para todos os testes foi de 0,05. A análise foi desenvolvida por profissional externo à pesquisa.

Os depoimentos foram analisados com Análise Textual Discursiva^23^, contemplando: unitarização, estabelecimento de relações e comunicação. Com esta análise, construiu-se uma categoria central que originou uma unidade de base e três categorias de análise.

Para a mixagem dos dados, utilizou-se a estratégia de incorporação dos dados qualitativos para complementar e aprofundar as análises do estudo experimental. Como recurso analítico, foram utilizados diagramas de exibição conjunta dos resultados das duas abordagens, bem como da combinação de ambas (*joint-display*)^24^.

### Aspectos éticos

Os participantes foram incluídos mediante ciência dos objetivos investigativos e assinatura de duas vias do Termo de Consentimento Livre e Esclarecido. Para o anonimato dos participantes, foram atribuídas as letras “B”, de bloco, e “P”, de participante, seguidas de algarismo alfanumérico (códigos B1P2, B1P4, B2P2 e B2P3 correspondem ao GI; códigos B1P1, B1P3, B2P1 e B2P4, ao GP). Após o término da coleta e análise de dados, os participantes do GP receberam orientações sobre sua condição e foi-lhes ofertado o protocolo interventivo pelo mesmo tempo e número de sessões do GI. Dois integrantes aceitaram receber os pontos intervenção.

Pesquisa aprovada por Comitê de Ética em Pesquisa, sob parecer n.º 3.897.861 e CAAE n.º 22328819.8.0000.5346, em março de 2020, e cadastrada no Registro Brasileiro de Ensaios Clínicos Randomizados (REBEc), obtendo o código RBR-3jvmdn.

## Resultados

Participaram do processo de randomização 27 profissionais de enfermagem, dos quais 13 foram alocados no GI e 14, no GP (Figura 1).

**Figura 1:** Fluxograma de alocação dos participantes do ensaio clínico randomizado, adaptado do *Consolidated Standards of Reporting Trials* ^15^. Santa Maria, RS, Brasil, 2022-2023 ^*^GI = Grupo intervenção; ^†^GP = Grupo placebo

A comparação entre as características do GI e do GP se apresentou com homogeneidade (p>0,05) (Tabela 1). 

**Tabela 1:** Caracterização dos participantes e prevalência de ansiedade e estresse nos grupos intervenção e controle (n = 27). Santa Maria, RS, Brasil, 2022-2023

Variáveis		Grupo Intervenção (n=13)		Grupo Placebo (n=14)		Valor de p
		n	%	n	%	
Estado civil	Casado	7	53,9	8	57,2	0,928*
	Solteiro	4	30,8	3	21,4	
	Divorciado	2	15,3	3	21,4	
Cargo	Enfermeiro(a)	5	38,5	3	21,4	0,645^†^
	Técnico(a)/ Auxiliar de enfermagem	8	61,5	11	78,6	
Intenção de deixar o cargo	Sim	2	15,4	2	14,3	0,546*
	Não	8	61,5	10	71,4	
	Talvez	3	23,1	2	14,3	
Ganho de peso nos últimos seis meses	Sim	7	53,8	9	64,3	0,704^†^
	Não	6	46,2	5	35,7	
Prática de atividade física	Sim	6	46,2	3	21,4	0,613*
	Não	5	38,5	6	42,9	
	Às vezes	2	15,3	5	35,7	
Possuem tempo para o lazer	Sim	3	23,0	4	28,6	0,851*
	Não	5	38,5	3	21,4	
	Às vezes	5	38,5	7	50,0	
Possuem problema de saúde diagnosticado	Sim	9	69,2	6	42,8	0,168^‡^
	Não	4	30,8	8	57,2	
		**Média**	**DP** ^§^	**Média**	**DP** ^§^	
Idade		41,75	8,92	43,38	6,88	0,611^||^
Prevalência de ansiedade antes da 1ª sessão		13,50	2,61	14,23	3,14	0,535^||^
Prevalência de estresse antes da 1ª sessão		77,58	16,64	82,69	32,03	0,626^||^

*Qui-quadrado com correção; ^†^Exato de Fisher; ^‡^Qui-quadrado; ^§^DP=Desvio-Padrão. ^||^Teste t de amostras independentes

Observou-se redução significativa de ansiedade e estresse intragrupos, entre os intervalos de sessões e *follow-up* (p<0,05). Não se observou redução entre os grupos (p>0,05) (Tabela 2). Os resultados da análise por intenção de tratar são semelhantes aos de protocolo.

**Tabela 2:** Comparação entre as médias de ansiedade e estresse dos grupos intervenção e placebo (análise de modelos mistos lineares, por protocolo), ao longo de quatro avaliações (n = 24). Santa Maria, RS, Brasil, 2022-2023

Desfecho	Grupo	Sessão (I)	Sessão (J)	Diferença de médias (I-J)	**Valor de *p***
Ansiedade*	Intervenção	1ª	4ª	4,96	**0,001**
			8ª	5,50	**0,004**
			*Follow-up*	5,30	**0,000**
		4ª	1ª	-4,96	**0,001**
			8ª	0,53	0,73
			*Follow-up*	0,33	0,82
		8ª	1ª	-5,50	**0,004**
			4ª	-0,54	0,73
			*Follow-up*	-0,19	0,88
		*Follow-up*	1ª	-5,30	**0,000**
			4ª	-0,34	0,82
			8ª	0,19	0,88
	Placebo	1ª	4ª	6,46	**0,000**
			8ª	5,16	**0,004**
			*Follow-up*	5,83	**0,000**
		4ª	1ª	-6,36	**0,000**
			8ª	-1,30	0,37
			*Follow-up*	-0,63	0,66
		8ª	1ª	-5,16	**0,004**
			4ª	1,30	0,37
			*Follow-up*	0,67	0,59
		*Follow-up*	1ª	-5,83	**0,00**
			4ª	0,63	0,66
			8ª	-0,67	0,59
Estresse^†^	Intervenção	1ª	4ª	14,72	**0,015**
			8ª	26,65	**0,002**
			*Follow-up*	17,50	**0,025**
		4ª	1ª	-14,72	**0,015**
			8ª	11,93	**0,016**
			*Follow-up*	2,78	0,59
		8ª	1ª	-26,65	**0,002**
			4ª	-11,93	**0,016**
			*Follow-up*	-9,14	0,09
		*Follow-up*	1ª	-17,50	**0,025**
			4ª	-2,78	0,59
			8ª	9,15	0,09
	Placebo	1ª	4ª	22,58	**0,000**
			8ª	27,00	**0,001**
			*Follow-up*	27,45	**0,001**
		4ª	1ª	-22,58	**0,000**
			8ª	4,41	0,30
			*Follow-up*	4,87	0,31
		8ª	1ª	-27,00	**0,001**
			4ª	-4,41	0,30
			*Follow-up*	0,45	0,93
		*Follow-up*	1ª	-27,45	**0,001**
			4ª	-4,87	0,32
			8ª	-0,46	0,93

Nota: As letras I e J indicam uma determinada sessão (I) em comparação com as demais (J)

*Mensurado com o instrumento Transtorno de Ansiedade Geral (GAD 7); ^†^Mensurado com a Lista de Sinais e Sintomas de Estresse (LSS)

Os dados qualitativos viabilizaram aprofundar a compreensão dos agravos investigados, possibilitando a construção da categoria central: “Auriculoterapia como intervenção para ansiedade e estresse”, unitarizada na unidade de base “Benefícios da auriculoterapia (pontos interventivos e placebo) para ansiedade e estresse de profissionais de enfermagem perioperatória” que, por sua vez, resultou nas categorias de análise: “Auriculoterapia para ansiedade e estresse com o grupo intervenção”; “Auriculoterapia para ansiedade e estresse com o grupo placebo”; e “Efeitos colaterais da auriculoterapia”.

### Auriculoterapia para ansiedade e estresse com o grupo intervenção

Antes da intervenção de auriculoterapia, os participantes apresentavam sintomas relacionados ao estresse e à ansiedade, como cansaço, esgotamento, dispneia, insônia, irritabilidade, falta e excesso de apetite:

*[...] antes da auriculoterapia eu me sentia irritada, estressada e esgotada. Exatamente assim* (B1P2).

*Estava bem mais irritada. [...] numa crise bem forte, ansiosa, estressada [...] havia momentos que eu não conseguia falar, eu tinha vontade de chorar [...] antes da pesquisa eu estava 10 em ansiedade, estresse e esgotamento, chegando no limite, não conseguia me desligar [...] eu comia aqui por obrigação n*ão é, eu faço hipoglicemia [...] tive vômito*, meu estômago doía, doía. Eu não dormia e nem comia em casa. O tempo inteiro com bruxismo, respiração superficial [...] estava exausta, eu tinha fome, mas eu não conseguia comer, eu estava, realmente, chegando no meu limite* (B1P4).

*Estresse, bastante estressado, principalmente de manhã cedo [...] descontava em casa. Era meio grosseiro nas respostas, ou até mesmo com os colegas. E ansiedade, sentia angústia, daí dava vontade de comer ou fumar* (B2P3).

Após serem submetidos à intervenção de auriculoterapia, pode-se evidenciar melhora/alívio dos sintomas de estresse e da ansiedade, com repercussões positivas na qualidade do sono e na diminuição do apetite e uso do tabaco. Os participantes referiram o desenvolvimento de estratégias de enfrentamento para os agravos, tais como enfrentar problemas respeitando os limites pessoais, a busca de relacionamento interpessoal com a equipe e de hábitos de vida saudáveis:

*[…] após a intervenção tenho me sentido bem melhor […] estou bem mais calma, mais tranquila, isso que o serviço está pesado […] o que está acontecendo após a intervenção é que os problemas no serviço continuam, só que agora não me afetam mais, vou tentando fazer o meu melhor sem interiorizar […] até o sono melhorou [...]* (B1P2).

*[…] ao longo da pesquisa, já na primeira semana, eu estava bem diferente, bem mais leve e calma, e foi bem gritante a mudança. As lesões já começaram a regredir, região cervical já não tinha mais nada, abdome, membros inferiores também já estavam regredindo. Comecei a me sentir melhor, já não sentia aquela angústia, aperto no peito, sufocada, aquela coisa de não conseguir falar [...] a ansiedade, eu posso dizer que chegou a zero, estou super calma e tranquila, com a cabeça descansada, o estresse não tem como zerar no ambiente de trabalho, mas numa escala de 10, está entre 1 e 2, o esgotamento também diminuiu, não me sinto mais esgotada, apenas um cansaço natural no dia [...]* (B1P4).

*Eu senti bastante melhora na ansiedade [...] estou fumando menos, emagreci bastante [...] quanto à irritação, até o pessoal está dizendo que eu estou mais tranquilo, falando menos, estou mais “zen”, e em casa também, não estou me estressando mais, estou tranquilo [...] eu acredito que a auriculoterapia fez efeito, no meu ponto de vista, me ajudou [...] eu comecei a me cuidar mais depois que eu fiz, estou me alimentando melhor, comecei a fazer a exercícios, voltei a jogar futebol, estou menos estressado [...] eu sentia muita dor no estômago ligada ao estresse. Antes eu não tinha persistência de fazer as coisas, aí depois que eu fiz, parece que fez uma lavagem cerebral na minha cabeça, e agora, eu tenho meta, estou conseguindo fazer* (B2P3).

Um participante não soube identificar se a melhora percebida ocorreu devido à intervenção ou pela redução dos estressores laborais.

*[...] não posso te dizer se melhorou ou piorou [...] eu não percebi muita diferença, porque eu também penso que aqui* [referindo-se ao serviço] *estamos num período muito turbulento [...]* (B2P2).

### Auriculoterapia para ansiedade e estresse com o grupo placebo

Os participantes, antes de receberem o placebo, referiram sintomas relacionados ao estresse e à ansiedade, os quais se manifestaram em aspectos físicos, psicológicos e comportamentais:

*Sentia-me bem ansiosa, há dias que realmente estou bem ansiosa […] a vida é corrida, então isso te gera bastante estresse, ansiedade, tanto no trabalho quanto na vida pessoal* (B1P1).

*Antes de receber, eu estava bem estressada, um pouco desmotivada também […] sentia taquicardia, sudorese, tinha pesadelo com o trabalho* (B1P3)*.*

*[…] as situações que acontecem no serviço acabam nos irritando […] eu sentia tristeza, acho que estava um pouco depressiva, comendo bastante, ansiosa, agora nos últimos dias, inclusive* (B2P1).

*Eu me sentia bastante nervosa, preocupada com várias coisas, com crises de ansiedade, preocupação com a sobrecarga de trabalho e tudo que eu tinha para fazer […] eu apresentava tremores, muita dor de cabeça, dor muscular, taquicardia, vertigens, muita vontade de chorar […]* (B2P4).

A seguir, apresenta-se a análise intergrupo de prevalência de ansiedade e estresse ao longo de quatro momentos de avaliação, dos participantes que completaram o protocolo interventivo (Tabela 3).

**Tabela 3:** Descritiva de média e desvio-padrão dos níveis ansiedade e estresse nos quatro momentos avaliados (n = 24). Santa Maria, RS, Brasil, 2022-2023

Desfecho	Grupo	n	Sessão 1	Sessão 4	Sessão 8	*Follow-up*
			Média (DP*)	Média (DP*)	Média (DP*)	Média (DP*)
Ansiedade	Intervenção	11	13,5(2,6)	8,7(4,7)	7,4(4,7)	9,4(4,1)
	Placebo	13	14,2(3,1)	9,0(5,5)	9,0(6,8)	7,6(5,1)
Estresse	Intervenção	11	77,5(16,6)	63,5(18,5)	51,2(27,9)	62,1(24,4)
	Placebo	13	82,6(32,0)	59,4(31,5)	54,9(32,2)	50,3(26,1)

*DP = Desvio-Padrão

Os depoimentos elucidam que, ao término das sessões da auriculoterapia com pontos placebos, alguns participantes não identificaram alívio/redução dos sintomas relacionados aos agravos estudados. Porém, houve relatos de melhora na disposição para o trabalho após as aplicações placebos:

*[…] eu não sei se fui placebo ou intervenção, mas até que agora estou um pouco mais tranquila, tentando fazer as coisas com mais calma, não fazer tudo correndo, porque não adianta […] eu até brincava que o meu* [acompanhamento de auriculoterpia] *era placebo, porque eu realmente estava bem ansiosa […] agora estou melhor* (B1P1).

*[...] durante a intervenção eu percebi uma melhora bem satisfatória, porque fiquei mais disposta para o trabalho, tranquila, menos ansiosa […] acredito que quando estava no período da aur*í*culo eu me sentia mais disposta no trabalho e pra tudo, agora parece que está faltando alguma coisa* (B1P3).

*Olha, o que que eu vou te dizer, eu não consegui notar […] mas as minhas colegas notaram* (B2P1).

*[…] Senti melhora, mas aí eu não sei se tem a ver com a auriculoterapia ou com a diminuição de responsabilidades que eu tinha […] eu não notei mudança no meu comportamento de saúde após as sessões de auriculoterapia* (B2P4).

### Efeitos colaterais da auriculoterapia

Os efeitos colaterais percebidos no GI e no GP foram autorrelatados como sintomas locais esperados pela aplicação da técnica, tais como desconforto, dor e/ou prurido por contato direto ou devido a pontos específicos.

*Só desconforto na hora de colocar. Local, não chega a ser dor, nada, só desconforto, nada demais* (B1P1).

*[…] um desconforto ao toque em alguns pontos, principalmente nos da parte interna da orelha, mas era ao toque. Dor, dor não, tinha dor se encostasse* (B1P2).

*[…] tive por momentos bastante desconforto […] estava me incomodando bastante, muita dor, coçava também, no lugar do ponto […] em alguns momentos, mas* não foi sempre (B1P3).

*Tiveram pontos doloridos, mas foi um ponto mais específico que era dolorido e foi regredindo com as trocas.* Não foi nada insuportável *[…] nada fora do comum […] não fiz nada, só esperei* [em relação à dor] (B1P4).

*[…] O único desconforto que eu senti foi na minha orelha esquerda que era mais sensível, daí eu preferia aplicações na direita […] é suportável* (B2P1).

*[…] quando tu esqueces e leva a mão […] alguns momentos que ficou mais “doloridinho”, alguns pontos, mas nada demais* (B2P2).

A Figura 2 apresenta as metainferências derivadas dos depoimentos no que diz respeito aos resultados quantitativos.

**Figura 2:** *Joint display* representativo da mixagem dos dados e metainferências

Resultados QUAN*	Resultados Qual^†^	Metainferências
A auriculoterapia apresentou redução significativa de ansiedade e estresse, na análise intragrupos, entre a 1ª e a 8ª sessão, e o *follow-up* (Tabela 1).	Unidade de base: “Benefícios da auriculoterapia (pontos interventivos e placebo) para ansiedade e estresse de profissionais de enfermagem perioperatória”.	Os profissionais de enfermagem perioperatória relataram sintomas expressivos de estresse e ansiedade previamente ao início do protocolo interventivo. A auriculoterapia apresentou resultados positivos na redução de estresse e ansiedade intragrupos, ou seja, tanto no grupo intervenção quanto no placebo, reforçando os benefícios do uso de práticas não farmacológicas.
No Grupo Intervenção, para a ansiedade, verificou-se diferença entre 1ª, 4ª (p^‡^=0,001), 8ª sessão (p^‡^=0,004) e *follow-up* (p^‡^=0,001); entre a 4ª e a 1ª (p^‡^=0,001); entre 8ª e 1ª sessão (p^‡^=0,004); e, entre *follow-up* e 1ª sessão (p^‡^=0,000). Quanto ao estresse, constataram-se diferenças entre 1ª, 4ª (p^‡^=0,015), 8ª sessão (p=0,002) e *follow-up* (p^‡^=0,025); entre a 4ª e a 1ª (p^‡^=0,015) e 8ª sessão (p^‡^=0,016); entre 8ª e 1ª (p^‡^=0,002) e a 4ª sessão (p^‡^=0,016); e, entre *follow-up* e 1ª sessão (p^‡^=0,025) (Tabela 2).	Categoria de análise: “Auriculoterapia para ansiedade e estresse com o grupo intervenção”.	A auriculoterapia apresentou-se benéfica para o controle da ansiedade e do estresse, porém, sem diferença estatística entre os grupos. Esta prática contribuiu para a melhora dos principais sintomas comuns a esses agravos (alterações no sono, no apetite, na irritabilidade e no uso de tabaco), bem como na promoção da autopercepção dos participantes.
		Os participantes relataram que passaram a utilizar estratégias de enfrentamento individuais para os estressores, após as sessões de auriculoterapia.
Obteve-se redução de 44,5% de ansiedade entre a 1ª e a 8ª sessão no Grupo Intervenção, sendo que no *follow-up* houve ligeiro aumento; no Grupo Placebo; uma redução de 36,75% no mesmo período foi constatada, mantendo-se no *follow-up*.	Categoria de análise: “Auriculoterapia para ansiedade e estresse com o grupo placebo”.	A estimulação verdadeira e de pontos sham (placebo) em auriculoterapia produziu efeitos fisiológicos ou relacionados à crença dos profissionais de enfermagem do grupo placebo, visto que relataram diminuição dos sintomas de ansiedade e estresse. Isso pode ser elucidado, visto que se tratam de agravos que, via de regra, respondem de forma positiva à escuta, à atenção dada ao indivíduo acometido. Ainda, um dos pontos utilizados no placebo foi o ponto tornozelo, que por mais que seja específico e utilizado quando se tem dor muscular nesse local, apresenta efeito analgésico, segundo a Medicina Tradicional Chinesa.
		A vivência da ansiedade e do estresse é singular, assim como os efeitos da auriculoterapia sobre as manifestações dos investigados é subjetiva. O sono, o apetite e outros sintomas foram regularizados em alguns participantes e, em outros, não. Ainda, essa prática atuou no coletivo, proporcionando equilíbrio emocional, redução da irritabilidade e melhor disposição para o trabalho.
Para o desfecho estresse, no Grupo Intervenção, houve redução de 34,01% de ansiedade entre a 1ª e a 8ª sessão, também não mantida no *follow-up*; quanto ao Grupo Placebo, entre a 1ª e a 8ª sessão, houve redução de 33,67%, com permanência de redução até o *follow-up* (Tabela 3).	Categoria de análise: “Efeitos colaterais da auriculoterapia”.	Auriculoterapia demonstrou-se minimamente invasiva, segura e com demanda de pouco tempo para a aplicação.
		Os eventos adversos vinculados à auriculoterapia foram mínimos. Foram autorreferidos sintomas locais, passageiros, toleráveis, que se manifestaram como desconforto no local da aplicação das semiagulhas, irritação na pele e hiperemia (vermelhidão).

*QUAN = Quantitativo; ^†^Qual = Qualitativo; ^‡^p=p valor

## Discussão

Constataram-se os efeitos benéficos da auriculoterapia em ambos os grupos para a redução de ansiedade e estresse, vivenciados por profissionais de enfermagem perioperatória. Aplicar auriculoterapia com uso de pontos verdadeiros ou *sham* (placebo) repercutiu em igual efeito sobre ansiedade e estresse dos investigados neste estudo. Observou-se melhora dos índices ao longo de oito sessões de auriculoterapia e no período de acompanhamento (*follow-up*).

A literatura é incipiente no que se refere a pesquisas de métodos mistos acerca da eficácia da auriculoterapia sobre os desfechos ansiedade e estresse, simultaneamente, vivenciados por profissionais de enfermagem perioperatória, dificultando comparações. Sob outra perspectiva, este panorama torna as evidências desta investigação pioneiras na temática em tela, visto que revela o potencial de incorporar dados qualitativos para explicar os efeitos de uma pesquisa experimental, características que proporcionam avanço em relação à literatura existente.

Estudo conduzido nos Estados Unidos da América evidenciou redução dos níveis de ansiedade (p<0,05) em profissionais da saúde que receberam auriculoterapia durante seis sessões^11^. No Brasil, profissionais da Atenção Básica tiveram níveis de estresse reduzidos após receberem a intervenção^25^. Investigação com profissionais de enfermagem em ambiente hospitalar durante a pandemia da COVID-19 constatou redução de ansiedade, estresse e depressão^26^.

Isso pode ser justificado em virtude do mecanismo de ação da auriculoterapia, o qual ocorre por meio da estimulação de áreas auriculares que estão associadas à formação reticular, a partir do sistema nervoso simpático e parassimpático. Tais informações são transmitidas do pavilhão auricular por meio de estímulo, através das fibras responsáveis pelas inervações. Entre os principais nervos, tem-se o nervo vago, responsável pelo envio de informações para regiões cerebrais essenciais na regulação da ansiedade^27^. 

No que se refere à evidência de que aplicar auriculoterapia com pontos verdadeiros ou placebo apresentou igual efeito nos níveis de ansiedade e estresse entre os profissionais investigados, outros estudos também constataram efeitos positivos com uso de placebo^10^ ^),(^ ^25^ ^),(^ ^28^. Quando utilizados para dor crônica na coluna vertebral^13^ e depressão^28^, pontos *sham* foram benéficos. Por outro lado, há evidências que comprovam que pontos interventivos específicos para determinados agravos repercutem em melhores efeitos, comparados ao placebo^10^ ^),(^ ^25^.

Nessa perspectiva, a estimulação de qualquer ponto auricular pode desencadear efeitos fisiológicos no organismo dos indivíduos, bem como produzir efeitos relacionados às suas crenças. Isso pode ser explicado com base em mecanismos neurológicos em que ocorre a liberação de neurotransmissores (ex. opioides, dopamina e serotonina), que ajustam as respostas biológicas dos pacientes; e mecanismos psicológicos, em que condições emocionais, como a ansiedade e controle pessoal, modificam a percepção do indivíduo sobre sua condição de saúde, aumentando sua resiliência^9^ ^),(^ ^13^ ^),(^ ^27^.

Cabe discutir acerca dos pontos utilizados nos grupos e seus efeitos, segundo a Medicina Tradicional Chinesa^9^. Os integrantes no GI receberam os pontos *shen men*, tronco cerebral, rim, simpático e fígado, evidenciados como os mais utilizados para ansiedade, estresse ou *burnout* ^10^. O ponto *shen men* possui efeito tranquilizante e analgésico, utilizado na ação excitatória e inibitória do córtex cerebral; o tronco cerebral estimula a mente, acalma o espírito, auxilia nas afecções do cérebro e é sedativo; o rim fortalece a energia essencial e a conservação da saúde; o simpático auxilia nas alterações circulatórias e neurovegetativas; e o ponto fígado regula emoções de irritabilidade, controla o *Qi* (energia) e ativa a circulação sanguínea^9^. 

Por sua vez, nos participantes do GP, bochecha, nariz externo, reto, tornozelo e traqueia foram os pontos auriculares aplicados, com funções de, respectivamente, tratar afecções faciais; aspectos da área do nariz, como inflamações, máculas, entre outras; tratamento de hemorroida interna e externa e prolapso retal; tratamento de entorse, inflamação da articulação; e acalmar a tosse, eliminar a fleuma e drenar a garganta^9^. Ou seja, condições que não estão diretamente relacionadas aos desfechos ansiedade e estresse. 

Nesse sentido, em um paralelo com a vertente francesa da auriculoterapia, aborda-se a teoria das zonas embriológicas, na qual a orelha é projetada em três membranas: ectoderme, mesoderme e endoderme. Associando os pontos utilizados neste estudo com essa teoria, percebe-se que o GI apresentou dois pontos vinculados à área mesodérmica, a qual é inervada pelos nervos occipital menor trigêmeo e auricular maior, e se vinculam a zonas de dores musculares e subcutâneas; também, à área endodérmica, inervada pelos nervos glossofaríngeo, facial, vago e trigêmeo, utilizados no tratamento de dores mais profundas^9^.

Sabe-se que existe relação entre os desfechos de estresse e dor. No contexto assistencial, estudo aborda que o estresse vivenciado pelos profissionais de enfermagem pode perpassar aspectos psicológicos e atingir questões físicas nos indivíduos, como a dor. Logo, a dor pode ser considerada um sinal de estresse, associada à ansiedade e tensões musculares^29^. Assim, considerando os pontos aplicados no GP, percebe-se que os participantes receberam estímulos em áreas relacionadas a dor, como o ponto tornozelo, o que pode ter contribuído para a melhora dos parâmetros de estresse e ansiedade entre os profissionais. 

No presente estudo, os intervencionistas eram enfermeiros ou estudantes de enfermagem, todos com curso de capacitação em auriculoterapia. No entanto, havia uma diferença no tempo de experiência dos terapeutas. Nesta perspectiva, um estudo clínico desenvolvido com estudantes portugueses constatou que a experiência e a prática clínica do profissional que está desenvolvendo a técnica são fundamentais para o desfecho clínico que está sendo avaliado^30^. Em virtude disso, foi desenvolvido um manual de intervenção para reduzir o viés e qualificar a capacitação dos terapeutas para desenvolver a técnica proposta^20^. 

Para além dos efeitos sobre a ansiedade e o estresse, a auriculoterapia contribuiu para a melhora do sono, controle de apetite, redução da irritabilidade e do uso de tabaco, bem como para a promoção da autopercepção dos participantes. Ainda, os profissionais passaram a utilizar estratégias de enfrentamento diante das adversidades laborais após as sessões de auriculoterapia. Destaca-se que esta prática, ao ser aplicada, visa o reestabelecimento do equilíbrio energético dos indivíduos, consequentemente, é benéfica à saúde^31^.

No que se refere aos efeitos colaterais identificados, verificou-se que, quando surgiram, apresentaram-se mínimos, pouco duradouros e como eventos locais. Este achado é reafirmado por pesquisa clínica que aponta que os eventos adversos relacionados à prática da auriculoterapia são ausentes ou pouco comuns^28^, evidenciando a segurança da prática. Porém, quando ocorrem, são transitórios e toleráveis, manifestando-se como desconforto e dor leve no local da aplicação^28^, irritação na pele e prurido^10^.

Diante do exposto, revela-se a importância da utilização de práticas não farmacológicas para a melhora de agravos emocionais, como ansiedade e estresse. A auriculoterapia demonstra-se uma prática simples, de boa aceitação, que repercute em efeitos positivos e benéficos para a saúde dos trabalhadores. Com os resultados deste estudo, percebe-se que os profissionais de enfermagem perioperatória se encontravam estressados e ansiosos e tais agravos se associaram às atividades dos seus contextos laborais. Desta forma, é essencial priorizar ações de promoção à saúde, promovendo a sua saúde psíquica, pois, ao passo que os trabalhadores se encontram saudáveis, uma assistência segura e qualificada é favorecida. 

Foram seguidas as diretrizes de condução para estudos experimentais e qualitativos. Porém, embora tenha havido capacitação e uso de manual, as sessões de auriculoterapia foram aplicadas por diferentes terapeutas e ocorreram casos de perda de pontos; também, o poder estatístico do desfecho estresse é baixo. Tais aspectos configuram limitações do estudo. Logo, as evidências desta pesquisa devem ser interpretadas e utilizadas com cautela.

Como contribuições para as áreas da saúde e da enfermagem, evidenciaram-se os benefícios da prática de auriculoterapia entre os trabalhadores de enfermagem perioperatória, para promoção da saúde e redução de níveis de estresse e ansiedade. Em suma, a auriculoterapia se apresentou como uma prática humana e integral ao indivíduo, visto que demonstrou benefícios para além dos desfechos primários. Assim, entende-se que as evidências desta pesquisa servem de subsídios para outras realidades poderem intervir com a auriculoterapia em favorecimento da saúde do trabalhador. 

## Conclusão

Constataram-se os benefícios da auriculoterapia para o alívio de ansiedade e estresse de profissionais de enfermagem perioperatória. A auriculoterapia aplicada com pontos verdadeiros e *sham* (placebo) resultou em igual efeito para a ansiedade e o estresse dos profissionais investigados. As evidências foram reforçadas com os depoimentos, os quais revelaram as contribuições acerca do uso da prática. Constatou-se a importância de intervenções não farmacológicas, como a auriculoterapia, a fim de ofertar subsídios para recuperação e promoção da saúde destes trabalhadores. 

Sugerimos que estudos futuros investiguem a eficácia da auriculoterapia em outras realidades e com demais categorias profissionais. Também, a comparação da técnica com diferentes materiais, como agulha semipermanente *versus* sementes ou cristais, é outra possibilidade de pesquisa. 

# Translation: Eficacia de la auriculoterapia para ansiedad y estrés en profesionales de enfermería perioperatoria: estudio mixto*

**DOI:** 10.1590/1518-8345.7218.4274

**Authors:** Oclaris Lopes Munhoz, Bruna Xavier Morais, Emanuelli Mancio Ferreira da Luz, Patrícia Bitencourt Toscani Greco, Silomar Ilha, Tânia Solange Bosi de Souza Magnago

**Affiliations:** 1 Universidade Federal do Rio Grande, Escola de Enfermagem, Rio Grande, RS, Brasil.; 2 Hospital da Brigada Militar de Santa Maria, Santa Maria, RS, Brasil.; 3 Universidade Federal de Santa Maria, Departamento de Ciências da Saúde, Palmeira das Missões, RS, Brasil.; 4 Universidade Federal de Santa Maria, Santa Maria, RS, Brasil.; 5 Becaria del Conselho Nacional de Desenvolvimento Científico e Tecnológico (CNPq), Brasil.

**Keywords:** Ansiedad, Estrés Laboral, Auriculoterapia, Enfermería Perioperatoria, Salud Laboral, Enfermería

## Abstract

**Objetivo:**

analizar la eficacia de la auriculoterapia en la ansiedad y estrés de profesionales de enfermería perioperatoria.

**Método:**

investigación de métodos mixtos, experimental e incorporada. En la etapa cuantitativa se realizó un ensayo clínico aleatorio, triple ciego, con profesionales de enfermería perioperatoria - que respondieron a un cuestionario de caracterización, a la Lista de Señales y Síntomas de Estrés, y al Trastorno de Ansiedad General GAD 7 - sometidos a ocho sesiones de auriculoterapia con agujas finas. La etapa cualitativa fue exploratoria y descriptiva, con datos obtenidos a través de entrevistas semiestructuradas. Datos mixtos con incorporación de los hallazgos cualitativos para examinar la intervención en el estudio experimental.

**Resultados:**

participaron 13 profesionales en el grupo intervención y 14 en el grupo control, habiéndose constatado reducción significativa de ansiedad y estrés intragrupos; sin embargo, sin diferencia estadística intergrupos (p>0,05). Datos cualitativos posibilitaron la construcción de la categoría central “Auriculoterapia como intervención para ansiedad y estrés”, que fue subdividida en una unidad de base y tres categorías, que versaron sobre los beneficios de la práctica.

**Conclusión:**

aplicar auriculoterapia con puntos verdaderos y falsos (*sham*) presentó igual efecto para ansiedad y estrés en los participantes investigados, evidencias que fueron reforzadas por las declaraciones. Intervenciones no farmacológicas, como la auriculoterapia, son importantes para recuperación y promoción de la salud de profesionales de enfermería perioperatoria. Registro Brasileño de Ensayos Clínicos: RBR-3jvmdn.

## Destacados

**(1)** Reducción significativa de ansiedad y estrés intragrupos, sin diferencia entre ellos.

**(2)** Auriculoterapia con puntos verdaderos y falsos (sham) presentó igual efecto.

**(3)** Estudio mixto, con datos cualitativos incorporados, para examinar una intervención.

**(4)** Datos pioneros de auriculoterapia, ansiedad y estrés en unidades perioperatorias.

**(5)** Intervenciones no farmacológicas son importantes en la promoción de la salud de trabajadores.

## Introducción

La evolución histórica de la salud del trabajador está impregnada por conquistas y beneficios dirigidos a mejores condiciones en los procesos laborales^1^. Por otro lado, existen ambientes en que todavía persisten factores que predispone a los trabajadores a enfermarse psíquicamente, como es el caso de instituciones de asistencia a la salud, siendo los más afectados los profesionales de enfermería^2^ ^)-(^ ^3^.

Las unidades en que ocurre el cuidado perioperatorio presentan condiciones que predisponen al desarrollo de ansiedad y estrés en profesionales de enfermería^3^ ^)-(^ ^4^. Estos trastornos pueden relacionarse al número limitado de equipos, a conflictos interprofesionales, y a la necesidad de conocimientos clínicos asistenciales específicos. Bloques quirúrgicos, salas de recuperación anestésica y centros de materiales y esterilización, representan sectores en los cuales la enfermería está más expuesta a la ansiedad y al estrés^3^ ^),(^ ^5^ ^)-(^ ^6^.

La ansiedad se caracteriza por miedo, sentimientos vagos y desagradables, generalmente relacionados a sufrimientos anticipados o a tensiones e incomodidades^7^. El estrés se relaciona a condiciones y demandas psicológicas a las que el individuo está expuesto, comprometiendo su salud psíquica^8^. Por norma, esas condiciones necesitan de cuidado terapéutico especializado, de uso de medicamentos y de estrategias de enfrentamiento^7^ ^)-(^ ^8^, aspectos que requieren adhesión, cambios de actitudes, condiciones sociales y tiempo disponible, características desafiadoras para el enfrentamiento de estos trastornos de la salud. Por tanto, se comprueba la importancia de las intervenciones alternativas.

En este contexto, la auriculoterapia es una práctica que beneficia la salud^9^ ^)-(^ ^13^. Se trata de una técnica milenaria que utiliza diferentes materiales esféricos o puntiagudos para estimular la oreja^9^, la que posee conexiones reflejo-somáticas vinculadas al Sistema Nervioso Central; una vez estimulados puntos específicos, la homeostasis es favorecida^9^. Además, es una práctica poco onerosa, con efectos colaterales mínimos/pasajeros, siendo que su aplicación lleva poco tiempo^9^ ^)-(^ ^10^.

Algunas investigaciones han revelado evidencias^10^ ^)-(^ ^13^ acerca del uso de la auriculoterapia, identificando eficacia para reducción de: dolor crónico en la columna vertebral^13^; ansiedad y estrés^10^ ^)-(^ ^11^. También participa en la promoción de la calidad de vida de profesionales de enfermería^12^. Sin embargo, investigaciones sobre la práctica, en los escenarios de cuidado perioperatorio - con medición concomitante de estos trastornos de salud y con métodos mixtos - son incipientes^10^. Así, la relevancia de esta investigación se muestra en los resultados analíticos del abordaje multimétodo, en donde los datos secundarios cualitativos explican los efectos de un experimento, produciendo inferencias más robustas, característica innovadora para estudios futuros. 

Considerando los vacíos de áreas ya mencionadas^10^, se objetivó analizar la eficacia de la auriculoterapia en la ansiedad y estrés en profesionales de enfermería perioperatoria.

## Método

### Delineamiento del estudio

Se trata de investigación de métodos mixtos, con uso de la estrategia experimental incorporada QUAN (cual)^14^, en la cual los datos cualitativos fueron incorporados para examinar el proceso de la intervención, en el estudio cuantitativo^14^. Un ensayo clínico aleatorio (ECA), triple ciego (paciente, estadístico y evaluadores de resultado), fue desarrollado en la etapa cuantitativa; una etapa cualitativa, exploratoria y descriptiva tuvo papel complementario.

Fueron seguidas las directrices CONSORT (*Consolidated Standards of Reporting Trials*)^15^, COREQ (*Consolidated Criteria for Reporting Qualitative Research*)^16^ y MMAT (*Mixed Methods Appraisal Tool*)^17^.

### Escenario del estudio

Se constituyeron los escenarios: Bloque quirúrgico (BC), Sala de Recuperación Anestésica (SRA), Unidad de Cirugía General (UCG) y Unidad de Procesamiento de Materiales y Esterilización (UPME), de un hospital de enseñanza en Rio Grande del Sur.

### Criterios de selección y muestra

Para el ECR, se constituyeron los criterios de inclusión: ser profesional de enfermería de las unidades arriba citadas; poseer disponibilidad para la realización de las sesiones de auriculoterapia; tener ansiedad (valores ≥ 10 son considerados positivos para la ocurrencia del trastorno de ansiedad generalizado)^18^; y, por lo menos, tener nivel medio de estrés (valores ≥ 29 puntos)^19^. Se excluyeron participantes con lesiones o inflamaciones en el pabellón auricular que comenzaron el uso de medicamento psicotrópico u otra terapia para el tratamiento de ansiedad y/o estrés, en el período del estudio o en hasta 30 días antes del inicio de la recolección de datos (objetivando evitar confusión de efectos). Participaron 74 profesionales de enfermería; 15 rechazaron participar y dos fueron excluidos por iniciar tratamiento para ansiedad. Así, 57 fueron evaluados como elegibles y 27 poseían los criterios de selección. 

En la etapa cualitativa, se incluyeron ocho profesionales participantes de la etapa experimental, por medio de sorteo: cuatro del grupo intervención (GI) y cuatro del placebo (GP).

### Recolección de datos

Los datos cuantitativos fueron recolectados entre octubre de 2022 y marzo de 2023. Los profesionales fueron convidados a responder a los instrumentos en el propio trabajo/unidad. Se aplicaron los instrumentos de investigación (suministrados a los participantes para que pudiesen responderlos) por evaluadores de resultados (recolectores), que no poseían conocimiento sobre la randomización y también eran responsables por evaluar si un determinado participante poseía los criterios de selección. Cuando los participantes atendían a los criterios establecidos, eran inmediatamente informados, convidados e incluidos en la intervención.

Los intervencionistas de auriculoterapia acompañaban ese proceso con los sobres (lacrados, opacos y numerados, consecutivamente) que contenían el grupo al cual el participante sería incluido. El sobre era abierto en la frente del participante, sin que este tuviese conocimiento sobre el grupo en que sería incluido. La randomización fue desarrollada en dos bloques (de 12 y 16 números, respectivamente), por investigador externo a la investigación, con el *Random Allocation Software* (RAS).

Después de la randomización, los participantes del GI y del GP fueron convidados a participar de las sesiones de aplicación de la técnica, individualmente. Cada profesional recibió ocho sesiones, dos por semana, con duración entre 10 y 15 minutos, en sus respectivos turnos de trabajo (mañana, tarde o noche, siendo los turnos mantenidos en las aplicaciones). Los participantes fueron evaluados en la 1ª, 4ª y al final de las 8 sesiones, y 15 días después del término de las aplicaciones (*follow-up*); el número de sesiones y acompañamientos fueron establecidos conforme metaanálisis previamente realizado^10^. Los cuestionarios de evaluación de resultado fueron aplicados en esos momentos por los mismos recolectores. 

Se utilizaron los siguientes cuestionarios para recolectar de datos cuantitativos: instrumento de perfil (edad, estado civil, cargo, intención de dejar el empleo, aumento de peso en los últimos seis meses, práctica de actividad física, tiempo para ocio y enfermedad diagnosticada por médico); el GAD 7 Trastorno General de Ansiedad, que evalúa ansiedad generalizada considerando las últimas dos semanas, posee siete ítems en escala Likert, con puntuaciones de 0 (ninguna vez), 1 (varios días), 2 (más de la mitad de los días) y 3 (casi todos los días)^18^. Ya, la Lista de Señales y Síntomas de Estrés (LSS) posee 60 ítems, señala la frecuencia con que se percibe los síntomas de estrés, con opciones de: 0 (nunca), 1 (raramente), 2 (frecuentemente) y 3 (siempre)^19^.

En lo que se refiere al protocolo de aplicación de la auriculoterapia por grupo, las aplicaciones fueron realizadas por cuatro terapeutas capacitados y con formación para la técnica (dos poseían cinco años de experiencia y otros dos un año). Los puntos seleccionados para los grupos intervención y placebo fueron definidos con base en la literatura^10^: para el GI, *shen men*, tronco cerebral, riñón, simpático e hígado (con efectos sedativos, analgésicos, ansiolíticos, sobre emociones y equilibrio)^9^; para el GP, se utilizaron los puntos mejilla, nariz externa, recto, tobillo y tráquea (cada uno con indicación específica para intervenir sobre alteraciones relacionadas a la región anatómica definida)^9^.

Para la detección de los puntos se utilizó un aparato de electro diagnóstico, marca *EL30 Finder Basic-NKL;* se objetivaba obtener mejor precisión de los puntos del GI. Para el GP, se buscó el área con menor respuesta al estímulo^20^. Además, se procedió con la antisepsia del pabellón auricular, con pinza anatómica envuelta en algodón embebido en alcohol 70%. Después, se aplicaron las agujas semipermanentes, marca *X Press*, tamaño 0,18 x 1,4 mm, posteriormente cubiertas por esparadrapo redondo hipoalergenico. Los participantes fueron orientados acerca de los cuidados necesarios con la práctica^20^. Cuatro participantes (tres del GI y uno del GP) informaron la pérdida de un punto auricular en una de las sesiones recibidas y que no trataron de recolocar la aguja fina, conforme orientación. También, tres integrantes del GI y dos del GP relataron dolor intensa en el primer día de aplicación, en dos sesiones distintas, pero optaron por no informar el intervencionista, ya que el síntoma se redujo entre el final del día de aplicación y el posterior. Ocurrieron otros tres casos de prurito local.

Los datos cualitativos fueron recolectados entre enero y marzo de 2023. Después del establecimiento de los participantes para esta recolección (conforme criterios ya mencionados anteriormente), se procedió a convidar a los profesionales a responder a la entrevista semiestructurada, en sus locales de trabajo. No hubo rechazos em esa participación.

Un manual específico fue desarrollado por el equipo y luego comprobado con dos integrantes del grupo de investigación. Se incluyeron las preguntas: ¿Cómo usted se sentía antes de recibir la intervención con auriculoterapia?; ¿Cómo usted se sintió o se siente después de la intervención?; ¿Usted notó algún cambio en su salud o comportamiento cotidiano después de las sesiones de auriculoterapia?; y, ¿Usted tuvo alguna incomodidad o algo semejante relacionado con la auriculoterapia?

Un investigador con doctorado en enfermería y experiencia en la técnica, y una discente de graduación en enfermería condujeron las entrevistas, en salas reservadas y libres de ruidos. Las declaraciones fueron grabadas en audio y tuvieron una duración media de 23 minutos. Para las transcripciones completas, se utilizó el *Microsoft Office Word* ^*®*^ . Dos integrantes del estudio revisaron las declaraciones, siendo realizadas adecuaciones necesarias de lenguaje. La saturación teórica^21^ fue considerada cuando se identificó repetición de los aspectos relacionados a las contribuciones de la auriculoterapia.

### Tratamiento y análisis de los datos

Los datos cuantitativos fueron introducidos en el programa Excel, de manera doble-independiente, por personal previamente capacitado, con posterior chequeo de las inconsistencias. Después fueron procesados con el *Statistical Package for the Social Sciences* (SPSS), versión 18.0. Las variables referentes al perfil del equipo fueron presentadas con frecuencias absolutas y relativas o media y desviación estándar.

El GAD-7 y la LSS fueron analizados con la suma de puntos de las preguntas. Para ansiedad, valores ≥ 10 indican trastorno de ansiedad generalizada presente; además, se puede categorizar en ansiedad mínima (0 a 4 puntos), baja (de 5 a 9), moderada (de 10 a 14) y severa (de 15 a 21 puntos)^18^. El estrés fue clasificado como ausente (de 0 a 11 puntos), bajo (de 12 a 28), medio (29 a 60), alto (61 a 120) y altísimo nivel (arriba de 120 puntos)^19^.

Para el análisis del resultado ansiedad, se consideró la inclusión de 13 participantes en el grupo intervención y 14 en el placebo. En un estudio anterior^10^, la respuesta dentro de cada grupo fue normalmente distribuida con desviación estándar 5,58. Considerando una diferencia clínicamente relevante de ansiedad entre los grupos de 7,14; un poder de 0,87 fue calculado para la muestra de este estudio. Para el estrés, la respuesta dentro de cada grupo fue normalmente distribuida con desviación estándar 36,36^10^; siendo la base una diferencia clínicamente relevante de 24,5; el poder calculado para a muestra fue de 0,36. La probabilidad de error Tipo I asociada al test de hipótesis nula fue de 0,05.

Para evaluación de homogeneidad de los grupos (características sociodemográficas, laborales, de salud, hábitos de vida y niveles iniciales de ansiedad y estrés), se utilizaron las pruebas Chi-cuadrado, Exacto de Fisher y Test t de muestras independientes.

En lo que se refiere a los datos de evaluación de resultados, correspondientes a la 1ª, 4ª y 8ª sesiones, y *follow-up*, se procedió con análisis de intención de tratar y de protocolo. Se utilizó de imputación múltiple para establecer datos ausentes en el tiempo. La comparación entre GI y GP fue realizada por medio de modelos lineares mixtos. Una estructura adecuada de covariancia (no estructurada) fue comprobada. Los parámetros del modelo fueron estimados por la probabilidad máxima^22^. El nivel de significación estadística para todas las pruebas fue de 0,05. El análisis fue desarrollado por profesional externo a la investigación.

Los declaraciones fueron analizadas con Análisis Textual Discursivo^23^, contemplando unitarización, establecimiento de relaciones y comunicación. Con este análisis, se construyó una categoría central que originó una unidad de base y tres categorías de análisis.

Para mezclar los datos, se utilizó la estrategia de incorporación de los datos cualitativos para complementar y profundizar los análisis del estudio experimental. Como recurso analítico, fueron utilizados diagramas de exhibición conjunta de los resultados de los dos abordajes, así como de la combinación de ambos (*joint-display*)^24^.

### Aspectos éticos

Los participantes fueron incluidos mediante la ciencia de los objetivos investigativos y firma de dos documentos del Término de Consentimiento Libre e Informado. Para el anonimato de los participantes, fueron atribuidas las letras “B”, de bloque, y “P”, de participante, seguidas de alegorismo alfanumérico (códigos B1P2, B1P4, B2P2 y B2P3 corresponden al GI; códigos B1P1, B1P3, B2P1 y B2P4, al GP). Después del término de la recolección y análisis de datos, los participantes del GP recibieron orientaciones sobre su condición y se ofreció el protocolo de intervención por el mismo tiempo y número de sesiones del GI. Dos integrantes aceptaron recibir los puntos intervención.

La investigación fue aprobada por el Comité de Ética en Investigación, con el número 3.897.861 y CAAE Nº 22328819.8.0000.5346, en marzo de 2020, e inscrita en el Registro Brasileño de Ensayos Clínicos Randomizados (REBEc), obteniendo el código RBR-3jvmdn.

## Resultados

Participaron del proceso de randomización 27 profesionales de enfermería, de los cuales 13 fueron asignados en el GI y 14, en el GP (Figura 1).

**Figura 1:** Diagrama de flujo de asignación de los participantes del ensayo clínico aleatorio, adaptado del *Consolidated Standards of Reporting Trials* ^15^. Santa Maria, RS, Brasil, 2022-2023 *GI = Grupo intervención; ^†^GP = Grupo placebo

La comparación entre las características del GI y del GP se presentó con homogeneidad (p>0,05) (Tabla 1).

**Tabla 1:** Caracterización de los participantes y prevalencia de ansiedad y estrés en los grupos intervención y control (n = 27). Santa Maria, RS, Brasil, 2022-2023

Variables		Grupo Intervención (n=13)		Grupo Placebo (n=14)		Valor de p
		n	%	n	%	
Estado civil	Casado	7	53,9	8	57,2	0,928*
	Soltero	4	30,8	3	21,4	
	Divorciado	2	15,3	3	21,4	
Cargo	Enfermero(a)	5	38,5	3	21,4	0,645^†^
	Técnico(a)/ Auxiliar de enfermería	8	61,5	11	78,6	
Intención de dejar el cargo	Si	2	15,4	2	14,3	0,546*
	No	8	61,5	10	71,4	
	Tal vez	3	23,1	2	14,3	
Aumento de peso en los últimos seis meses	Si	7	53,8	9	64,3	0,704^†^
	No	6	46,2	5	35,7	
Práctica de actividad física	Si	6	46,2	3	21,4	0,613*
	No	5	38,5	6	42,9	
	A veces	2	15,3	5	35,7	
Poseen tiempo para el ocio	Si	3	23,0	4	28,6	0,851*
	No	5	38,5	3	21,4	
	A veces	5	38,5	7	50,0	
Poseen problemas de salud diagnosticado	Si	9	69,2	6	42,8	0,168^‡^
	No	4	30,8	8	57,2	
		**Media**	**DE** ^§^	**Media**	**DE** ^§^	
Edad		41,75	8,92	43,38	6,88	0,611^||^
Prevalencia de ansiedad antes de la 1ª sesión		13,50	2,61	14,23	3,14	0,535^||^
Prevalencia de estrés antes de la 1ª sesión		77,58	16,64	82,69	32,03	0,626^||^

*Chi-cuadrado con corrección; ^†^Exacto de Fisher; ^‡^Chi-cuadrado; ^§^DE = Desviación Estándar. ^||^Test t de muestras independientes

Se observó reducción significativa de ansiedad y estrés intragrupos, entre los intervalos de sesiones y *follow-up* (p<0,05). No se observó reducción entre los grupos (p>0,05) (Tabla 2). Los resultados del análisis por intención de tratar son semejantes a los del protocolo. 

**Tabla 2:** Comparación entre las medias de ansiedad y estrés de los grupos intervención y placebo (análisis de modelos mixtos lineares, por protocolo), a lo largo de cuatro evaluaciones (n = 24). Santa Maria, RS, Brasil, 2022-2023

Resultado	Grupo	Sesión (I)	Sesión (J)	Diferencia de medias (I-J)	**Valor de *p***
Ansiedad*	Intervención	1ª	4ª	4,96	**0,001**
			8ª	5,50	**0,004**
			*Follow-up*	5,30	**0,000**
		4ª	1ª	-4,96	**0,001**
			8ª	0,53	0,73
			*Follow-up*	0,33	0,82
		8ª	1ª	-5,50	**0,004**
			4ª	-0,54	0,73
			*Follow-up*	-0,19	0,88
		*Follow-up*	1ª	-5,30	**0,000**
			4ª	-0,34	0,82
			8ª	0,19	0,88
	Placebo	1ª	4ª	6,46	**0,000**
			8ª	5,16	**0,004**
			*Follow-up*	5,83	**0,000**
		4ª	1ª	-6,36	**0,000**
			8ª	-1,30	0,37
			*Follow-up*	-0,63	0,66
		8ª	1ª	-5,16	**0,004**
			4ª	1,30	0,37
			*Follow-up*	0,67	0,59
		*Follow-up*	1ª	-5,83	**0,00**
			4ª	0,63	0,66
			8ª	-0,67	0,59
Estrés^†^	Intervención	1ª	4ª	14,72	**0,015**
			8ª	26,65	**0,002**
			*Follow-up*	17,50	**0,025**
		4ª	1ª	-14,72	**0,015**
			8ª	11,93	**0,016**
			*Follow-up*	2,78	0,59
		8ª	1ª	-26,65	**0,002**
			4ª	-11,93	**0,016**
			*Follow-up*	-9,14	0,09
		*Follow-up*	1ª	-17,50	**0,025**
			4ª	-2,78	0,59
			8ª	9,15	0,09
	Placebo	1ª	4ª	22,58	**0,000**
			8ª	27,00	**0,001**
			*Follow-up*	27,45	**0,001**
		4ª	1ª	-22,58	**0,000**
			8ª	4,41	0,30
			*Follow-up*	4,87	0,31
		8ª	1ª	-27,00	**0,001**
			4ª	-4,41	0,30
			*Follow-up*	0,45	0,93
		*Follow-up*	1ª	-27,45	**0,001**
			4ª	-4,87	0,32
			8ª	-0,46	0,93

Nota: Las letras I y J indican una determinada sesión (I) en comparación con las demás (J)

*Medido con el instrumento Trastorno de Ansiedad General (GAD 7); ^†^Medido con la Lista de Señales y Síntomas de Estrés (LSS)

Los datos cualitativos permitieron profundizar la comprensión de los trastornos investigados, posibilitando la construcción de la categoría central: “Auriculoterapia como intervención para ansiedad y estrés”, unitarizada en la unidad de base “Beneficios de la auriculoterapia (puntos de intervención y placebo) para ansiedad y estrés de profesionales de enfermería perioperatoria” que, a su vez, resultó en las categorías de análisis: “Auriculoterapia para ansiedad y estrés con o grupo intervención”; “Auriculoterapia para ansiedad y estrés con o grupo placebo”; y, “Efectos colaterales de la auriculoterapia”.

### Auriculoterapia para ansiedad y estrés con el grupo intervención

Antes de la intervención de auriculoterapia, los participantes presentaban síntomas relacionados al estrés y ansiedad, como cansancio, agotamiento, dipnea, insomnio, irritabilidad y falta/exceso de apetito:

*[...] antes de la auriculoterapia yo me sentía irritada, estresada y agotada. Exactamente así* (B1P2).

*Estaba bien más irritada. [...] en una crisis bien fuerte, ansiosa, estresada [...] había momentos que yo no conseguía hablar, yo tenía ganas de llorar [...] antes de la investigación yo estaba 10 en ansiedad, estrés y agotamiento, llegando al l*í*mite, no consegu*í*a me desconectar [...] yo com*í*a aqu*í *por obligación ¿verdad?, yo tengo hipoglucemia [...] tuve vómito, mi est*ó*mago dol*ía, do*l*ía. *Yo no dorm*í*a y ni com*í*a en casa. El tiempo entero con bruxismo, respiración superficial [...] estaba exhausta, yo tenía hambre, pero yo no consegu*í*a comer, yo estaba, realmente, llegando en mi l*í*mite* (B1P4).

*Estr*é*s, bastante estresada, principalmente de ma*ñana *temprano [...] descargaba en casa. Era medio grosero en las respuestas, o hasta mismo con las compañeras. Y ansiedad, sent*í*a angustia, por eso sentía voluntad de comer o fumar* (B2P3).

Después de ser sometidos a la intervención de auriculoterapia, se pudo evidenciar mejoría/alivio de los síntomas de estrés y ansiedad, con repercusiones positivas en la calidad del sueño y en la disminución del apetito y uso del tabaco. Los participantes refirieron el desarrollo de estrategias de enfrentamiento para los trastornos: como enfrentar problemas respetando los límites personales, búsqueda de relacionamiento interpersonal con el equipo y hábitos de vida saludables:

*[…] después de la intervención me he sentido bien mejor […] estoy bien más calmada, más tranquila, eso que el servicio está pesado […] lo que está sucediendo después de la intervención es que los problemas en el servicio continúan, pero ahora no me afectan más, voy tratando de hacer lo mejor sin interiorizar […] hasta el sueño mejor*ó *[...]* (B1P2).

*[…] a lo largo de la investigación, ya en la primera semana, yo estaba bien diferente, bien más liviana y calmada, y fue bien fuerte el cambio. Las lesiones ya comenzaron a retroceder, regi*ón *cervical ya no tenía más nada, abdomen, miembros inferiores también ya estaban retrocediendo. Comenc*é *a sentirme mejor, ya no sent*í*a aquella angustia, aprieto en el pecho, sofocada, aquella cosa de no conseguir hablar [...] la ansiedad, yo puedo decir que llegó a cero, estoy muy calmada y tranquila, con la cabeza descansada, el estrés no tiene como ser cero en el ambiente de trabajo, pero en una escala de 10, está entre 1 y 2, el agotamiento también disminuyó, no me siento más agotada, apenas un cansancio natural en el* día *[...]* (B1P4).

*Yo sent*í *bastante mejor*í*a en la ansiedad [...] estoy fumando menos, adelgace bastante [...] En lo que se refiere a la irritación, hasta el personal está diciendo que yo estoy más tranquilo, hablando menos, estoy más “zen”, y en casa también, no estoy estresándome más, estoy tranquilo [...] yo creo que la auriculoterapia hace efecto, en mi punto de vista, me ayudó [...] yo comencé a cuidarme más después que yo hice, estoy aliment*á*ndome mejor, comenc*é *a hacer a ejercicios, volví a jugar futbol, estoy menos estresado [...] yo sentía mucho dolor en el est*ó*mago proveniente del estrés. Antes yo no tenía persistencia de hacer las cosas, después que yo hice, parece que hizo un lavado cerebral en mi cabeza, y ahora, yo tengo meta, estoy consiguiendo hacer* (B2P3).

Um participante no pudo identificar si la mejoría percibida ocurrió debido a la intervención o por la reducción de los estresores laborales.

*[...] no te puedo decir si mejor*ó *o empeoró [...] yo no percibí mucha diferencia, porque yo también pienso que aqu*í [refiriéndose al servicio] *estamos en un período muy turbulento [...]* (B2P2).

### Auriculoterapia para ansiedad y estrés en el grupo placebo

Los participantes, antes de recibir el placebo, refirieron síntomas relacionados al estrés y a la ansiedad, los que se manifestaron en aspectos físicos, psicológicos y comportamentales:

*Me sent*í*a bien ansiosa, hace* días *que realmente estoy bien ansiosa […] la vida es corrida, entonces eso te genera bastante estrés, ansiedad, tanto en el trabajo cono en la vida personal* (B1P1).

*Antes de recibir, yo estaba bien estresada, un poco desmotivada también […] sent*í*a taquicardia, sudorese, tenía pesadillas con el trabajo* (B1P3).

*[…] las situaciones que suceden en el servicio acaban nos irritando […] yo sent*í*a tristeza, creo que estaba un poco depresiva, comiendo bastante, ansiosa, ahora en los* últimos días*, inclusive* (B2P1).

*Yo me sent*í*a bastante nerviosa, preocupada con varias cosas, con crisis de ansiedad, preocupación con la sobrecarga de trabajo y todo lo que yo tenía para hacer […] yo presentaba tremores, mucho dolor de cabeza, dolor muscular, taquicardia, vértigos, mucha ganas de llorar […]* (B2P4).

A seguir, se presenta el análisis intergrupo de prevalencia de ansiedad y estrés a lo largo de cuatro momentos de evaluación, de los participantes que completaron el protocolo de intervención (Tabla 3).

**Tabla 3:** Descripción de la media y desviación estándar de los niveles ansiedad y estrés en los cuatro momentos evaluados (n = 24). Santa Maria, RS, Brasil, 2022-2023

Resultado	Grupo	n	Sesión 1	Sesión 4	Sesión 8	*Follow-up*
			Media (DE*)	Media (DE*)	Media (DE*)	Media (DE*)
Ansiedad	Intervención	11	13,5(2,6)	8,7(4,7)	7,4(4,7)	9,4(4,1)
	Placebo	13	14,2(3,1)	9,0(5,5)	9,0(6,8)	7,6(5,1)
Estrés	Intervención	11	77,5(16,6)	63,5(18,5)	51,2(27,9)	62,1(24,4)
	Placebo	13	82,6(32,0)	59,4(31,5)	54,9(32,2)	50,3(26,1)

*DE = Desviación Estándar

Las declaraciones elucidan que, al término de las sesiones de la auriculoterapia con puntos placebos, algunos participantes no identificaron alivio/reducción de los síntomas relacionados a los síntomas estudiados. Sin embargo, hubo relatos de mejoría en la disposición para el trabajo después de las aplicaciones en los placebos:

*[…] yo no s*é *si fui placebo o intervención, pero hasta que ahora estoy un poco más tranquila, tratando de hacer las cosas con más calma, no hacer todo corriendo, porque no sirve […] yo hasta jugaba que yo* [acompañamiento de auriculoterapia] *era placebo, porque yo realmente estaba bien ansiosa […] ahora estoy mejor* (B1P1).

*[...] durante la intervención yo percib*í *una mejoría bien satisfactoria, porque estuve más dispuesta para el trabajo, tranquila, menos ansiosa […] creo que cuando estaba en el período de la auriculoterapia yo me sent*í*a más dispuesta en el trabajo y para todo, ahora parece que está faltando alguna cosa* (B1P3).

*Vea, lo que yo voy a decirte, yo no consegu*í *notar […] pero mis compañeras notaron* (B2P1).

*[…] Sent*í *mejoría, pero ah*í *yo no s*é *si tiene que ver con la auriculoterapia o con la disminución de responsabilidades que yo tenía […] yo no not*é *cambios en mi comportamiento de salud, después de las sesiones de auriculoterapia* (B2P4).

### Efectos colaterales de la auriculoterapia

Los efectos colaterales percibidos en el GI y en el GP fueron relatados como síntomas locales esperados por la aplicación de la técnica, tales como incomodidad, dolor y/o prurito por contacto directo o debido a puntos específicos.

*Solo incomodidad en la hora de colocar. Local, no llega a ser dolor, nada, solo incomodidad, nada demás* (B1P1).

*[…] una incomodidad al toque en algunos puntos, principalmente en los de la parte interna de la oreja, pero era al toque. Dolor, dolor no, tenía dolor si me acercase* (B1P2).

*[…] tuve por momentos bastante incomodidad […] estaba me incomodando bastante, mucho dolor, picaba también, en el lugar del punto […] en algunos momentos, pero no fue siempre* (B1P3).

*Hubo puntos doloridos, pero fue un punto más específico que causaba dolor y fue disminuyendo con los cambios. No fue nada insoportable […] nada fuera de lo com*ún *[…] no hice nada, solo esper*é [en relación al dolor] (B1P4).

*[…] La* únic*a incomodidad que yo sent*í *fue en mi oreja izquierda que era más sensible, por eso yo prefer*í *aplicaciones en la derecha […] es soportable* (B2P1).

*[…] cuando tu olvidas y llevas la mano […] algunos momentos que quedó más “dolorida”, algunos puntos, pero nada demás* (B2P2).

La Figura 2 presenta las metainferencias derivadas de las declaraciones en lo que se refiere a los resultados cuantitativos.

**Figura 2:** *Joint display* representativo de la mezcla de los datos y metainferencias

Resultados QUAN*	Resultados Qual^†^	Metainferencias
La auriculoterapia presentó reducción significativa de ansiedad y estrés, en el análisis intragrupos, entre la 1ª y 8ª sesión, y el *follow-up* (Tabla 1).	Unidad de base: “Beneficios de la auriculoterapia (puntos de intervención y placebo) para ansiedad y estrés de profesionales de enfermería perioperatoria”.	Los profesionales de enfermería perioperatoria relataron síntomas expresivos de estrés y ansiedad antes del inicio del protocolo de intervención. La auriculoterapia presentó resultados positivos en la reducción de estrés y ansiedad intragrupos, o sea, tanto en el grupo de intervención como en el placebo, reforzando los beneficios del uso de prácticas no farmacológicas.
En el Grupo Intervención, para la ansiedad, se verificó diferencia entre 1ª, 4ª (p^‡^=0,001), 8ª sesión (p^‡^=0,004) y *follow-up* (p^‡^=0,001); entre la 4ª y 1ª (p^‡^=0,001); entre 8ª y 1ª sesión (p^‡^=0,004); y, entre *follow-up* y 1ª sesión (p^‡^=0,000). Referente al estrés, se constataron diferencias entre 1ª, 4ª (p^‡^=0,015), 8ª sesión (p=0,002) y *follow-up* (p^‡^=0,025); entre la 4ª y la 1ª (p^‡^=0,015) y 8ª sesión (p^‡^=0,016); entre 8ª y 1ª (p^‡^=0,002) y la 4ª sesión (p^‡^=0,016); y, entre *follow-up* y 1ª sesión (p^‡^=0,025) (Tabla 2).	Categoría de análisis: “Auriculoterapia para ansiedad y estrés con el grupo intervención”.	La auriculoterapia se presentó benéfica para el control de la ansiedad y del estrés; sin embargo, sin diferencia estadística entre los grupos. Esta práctica contribuyó para la mejoría de los principales síntomas comunes a esos trastornos (alteraciones en el sueño, en el apetito, en la irritabilidad y en el uso de tabaco), así como en la promoción de la autopercepción de los participantes.
		Los participantes relataron que pasaron a utilizar estrategias de enfrentamiento individuales para los estresores, después de las sesiones de auriculoterapia.
Se obtuvo reducción de 44,5% de ansiedad entre la 1ª y la 8ª sesión en el Grupo Intervención, siendo que en el *follow-up* hubo ligero aumento; en el Grupo Placebo, una reducción de 36,75% en el mismo período fue constatada, manteniéndose en el *follow-up*.	Categoría de análisis: “Auriculoterapia para ansiedad y estrés con el grupo placebo”.	La estimulación verdadera y de puntos *sham* (placebo) en auriculoterapia produjo efectos fisiológicos o relacionados a la creencia de los profesionales de enfermería del grupo placebo, considerando que relataron disminución de los síntomas de ansiedad y estrés. Eso puede ser elucidado, al considerar que se trata de trastornos que, generalmente, responden de forma positiva a la escucha, en la atención dada al individuo afectado. Además, uno de los puntos utilizados en el placebo fue el punto tobillo, que por más que sea específico y utilizado cuando se tiene dolor muscular en ese local, presenta efecto analgésico, según la Medicina Tradicional China.
		La experiencia de la ansiedad y del estrés es singular, así como los efectos de la auriculoterapia sobre las manifestaciones de los investigados es subjetiva. El sueño, el apetito y otros síntomas fueron regularizados en algunos participantes y, en otros no. También, esa práctica actuó sobre el colectivo, proporcionando equilibrio emocional, reducción de la irritabilidad y mejor disposición para el trabajo.
Para el resultado estrés, en el Grupo Intervención, hubo reducción de 34,01% de ansiedad entre a 1ª y a 8ª sesión, también no mantenida en el *follow-up.* El Grupo Placebo, entre la 1ª y la 8ª sesión, presentó reducción de 33,67%, con permanencia de reducción hasta el *follow-up* (Tabla 3).	Categoría de análisis: “Efectos colaterales de la auriculoterapia”.	La auriculoterapia se demostró muy poco invasora, segura y utilización de poco tiempo para la aplicación.
		Los eventos adversos vinculados a la auriculoterapia fueron mínimos. Fueron autorreferidos síntomas locales, pasajeros, tolerables, que se manifestaron como incomodidad en el local de la aplicación de las agujas finas, irritación en la piel e hiperemia (enrojecimiento).

*QUAN = Cuantitativo; ^†^QUAL = Cualitativo; ^‡^p = p valor

## Discusión

Se constataron los efectos benéficos de la auriculoterapia, en ambos grupos, para la reducción de ansiedad y estrés, experimentados por profesionales de enfermería perioperatoria. Aplicar auriculoterapia con uso de puntos verdaderos o *sham* (placebos) repercutió con igual efecto sobre ansiedad y estrés en los investigados de este estudio. Se observó mejoría de los índices a lo largo de ocho sesiones de auriculoterapia y en el período de acompañamiento (*follow-up*).

La literatura es incipiente en lo que se refiere a investigaciones de métodos mixtos acerca de la eficacia de la auriculoterapia - sobre los resultados ansiedad y estrés, simultáneamente, experimentados por profesionales de enfermería perioperatoria - lo que dificulta las comparaciones. Bajo otra perspectiva, este panorama torna las evidencias de esta investigación pioneras en la presente temática, ya que revela el potencial de incorporar datos cualitativos, para explicar los efectos de una investigación experimental, característica que proporciona avances en relación a la literatura existente.

Un estudio realizado en Estados Unidos evidenció reducción de los niveles de ansiedad (p<0,05) en profesionales de la salud que recibieron auriculoterapia durante seis sesiones^11^. En Brasil, los profesionales de la Atención Básica tuvieron niveles de estrés reducidos después de recibir la intervención^25^. Una investigación con profesionales de enfermería en ambiente hospitalario durante la pandemia de la Covid-19 constató reducción de ansiedad, estrés y depresión^26^. 

Eso puede ser justificado en virtud del mecanismo de acción de la auriculoterapia, que ocurre por medio de la estimulación de áreas auriculares que están asociadas a la formación reticular, a partir del sistema nervoso simpático y parasimpático; esas informaciones son transmitidas del pabellón auricular por medio de estímulo, a través de las fibras responsables por las inervaciones. Entre los principales nervios, tenemos el nervio vago, responsable por el envío de informaciones para regiones cerebrales esenciales para la regulación de la ansiedad^27^. 

En lo que se refiere a la evidencia sobre la aplicación de auriculoterapia con puntos verdaderos o placebos, esta presentó igual efecto en los niveles de ansiedad y estrés, entre los profesionales investigados. Otros estudios también constataron efectos positivos con uso de placebo^10^ ^),(^ ^25^ ^),(^ ^28^. Cuando utilizados para dolor crónico en la columna vertebral^13^ y depresión^28^, los puntos *sham* fueron benéficos. Por otro lado, existen evidencias que comprueban que puntos de intervenciones específicos para determinados síntomas perjudiciales repercuten en mejores efectos, cuando comparados con el placebo^10^ ^),(^ ^25^.

En esa perspectiva, la estimulación de cualquier punto auricular puede desencadenar efectos fisiológicos en el organismo de los individuos, así como producir efectos relacionados a sus creencias. Eso puede ser explicado: 1) con base en mecanismos neurológicos en los que ocurre la liberación de neurotransmisores (opioides, dopamina y serotonina), que ajustan las respuestas biológicas de los pacientes, y 2) por mecanismos psicológicos, que contienen condiciones emocionales, como la ansiedad y el control personal, los que modifican la percepción del individuo sobre su condición de salud y aumentan su resiliencia ^9^ ^),(^ ^13^ ^),(^ ^27^.

Cabe discutir acerca de los puntos utilizados en los grupos y sus efectos, según la Medicina Tradicional China^9^. Los integrantes del GI recibieron los puntos *shen men*, tronco cerebral, riñón, simpático e hígado, evidenciados como los más utilizados para ansiedad, estrés ou *burnout* ^10^. El punto *shen men* posee efecto tranquilizante y analgésico, utilizado en la acción de excitación e inhibidora de la corteza cerebral: el tronco cerebral estimula la mente, calma el espíritu, auxilia en las enfermedades del cerebro y es sedativo; el riñón fortalece la energía esencial y la conservación de la salud; el simpático auxilia en las alteraciones circulatorias y neurovegetativas; y, el punto hígado regula emociones de irritabilidad, controla el *Qi* (energía) y activa la circulación sanguínea^9^. 

A su vez, en los participantes del GP, la mejilla, nariz externa, recto, tobillo y tráquea fueron los puntos auriculares aplicados, con funciones de, respectivamente: tratar trastornos faciales, aspectos del área de la nariz, como inflamaciones, máculas, entre otras; tratamiento de hemorroides interna y externa y prolapso rectal; tratamiento de esguince, inflamación de la articulación; y, calmar la tos, eliminar la flema y drenar la garganta^9^. O sea, condiciones que no están directamente relacionadas con los resultados ansiedad y estrés. 

En ese sentido, en un paralelo con la vertiente francesa de la auriculoterapia, se aborda la teoría de las zonas embriológicas, en la cual la oreja es proyectada en tres membranas: ectodermo, mesodermo y endodermo. Asociando los puntos utilizados en este estudio con esa teoría, se percibe que el GI presentó dos puntos vinculados al área mesodérmica, la que es inervada por los nervios occipital menor trigémino y auricular mayor, y se vinculan a zonas de dolores musculares y subcutáneas; también, el área endodérmica, inervada por los nervios glosofaríngeo, facial, vago y trigémino, utilizados en el tratamiento de dolores más intensas^9^.

Se sabe que existe relación entre los resultados de estrés y dolor. En el contexto asistencial, un estudio mostró que el estrés experimentado por los profesionales de enfermería puede relacionarse con aspectos psicológicos y producir efectos físicos en los individuos, como el dolor. De tal modo que, el dolor puede ser considerado una señal de estrés, asociada a la ansiedad y tensiones musculares^29^. Así, considerando los puntos aplicados en el GP, se percibe que los participantes recibieron estímulos en áreas relacionadas al dolor, como el punto tobillo, lo que pudo haber contribuido para la mejoría de los parámetros de estrés y ansiedad, entre los profesionales. 

En este estudio, los intervencionistas eran enfermeros o estudiantes de enfermería, todos con curso de capacitación en auriculoterapia. Sin embargo, había una diferencia en el tiempo de experiencia de los terapeutas. Dentro de esta perspectiva, un estudio clínico desarrollado con estudiantes portugueses constató que la experiencia y la práctica clínica del profesional, que está desarrollando la técnica, son fundamentales para el resultado clínico que está siendo evaluado^30^. En virtud de eso, fue desarrollado un manual de intervención para reducir el sesgo y calificar la capacitación de los terapeutas para desarrollar la técnica propuesta^20^. 

Yendo más allá de los efectos producidos sobre la ansiedad y el estrés, la auriculoterapia contribuyó para la mejoría del sueño, control de apetito, reducción de la irritabilidad y del uso de tabaco, así como para la promoción de la autopercepción de los participantes. Además, los profesionales, después de las sesiones de auriculoterapia, pasaron a utilizar estrategias de enfrentamiento delante de las adversidades laborales. Se destaca que esta práctica, al ser aplicada, tiene por objetivo el restablecimiento del equilibrio energético de los individuos, consecuentemente, es benéfica para la salud^31^.

En lo que se refiere a los efectos colaterales identificados, se verificó que, cuando surgieron fueron mínimos, duraron poco y eran eventos locales. Este hallazgo fue confirmado por investigación clínica que apuntó que los eventos adversos, relacionados a la práctica de la auriculoterapia, son ausentes o poco comunes^28^, evidenciando la seguridad de la práctica. Sin embargo, cuando ocurren, son transitorios y tolerables, manifestándose como incomodidad y dolor leve en el local de la aplicación^28^, irritación en la piel y prurito^10^.

Delante de lo expuesto, se revela la importancia de la utilización de prácticas no farmacológicas para la mejoría de trastornos emocionales, como ansiedad y estrés. La auriculoterapia demuestra ser una práctica simple, de buena aceptación, que repercute en efectos positivos y benéficos para la salud de los trabajadores. Con los resultados de este estudio, se percibió que los profesionales de enfermería perioperatoria se encontraban estresados y ansiosos y estos trastornos perjudiciales se asociaron con las actividades de sus contextos laborales. De esta forma, es esencial priorizar acciones de promoción a la salud, promoviendo la salud psíquica, considerando que si los trabajadores se encuentran saludables, una asistencia segura y calificada es favorecida. 

Fueron seguidas las directrices de conducción para estudios experimentales y cualitativos. Sin embargo, a pesar de haber sido dada la capacitación y usado el manual, las sesiones de auriculoterapia fueron aplicadas por diferentes terapeutas y ocurrieron casos de pérdida de puntos; también, el poder estadístico del resultado estrés fue bajo. Estos aspectos configuran limitaciones del estudio. Por tanto, las evidencias de esta investigación deben ser interpretadas y utilizadas con cautela.

Como contribuciones para las áreas de salud y enfermería, se evidenciaron los beneficios de la práctica de auriculoterapia, entre los trabajadores de enfermería perioperatoria, para promoción de la salud y reducción de niveles de estrés y ansiedad. En suma, la auriculoterapia se presentó como una práctica humana e integrada con el individuo, que demostró beneficios que van más allá de los resultados primarios. Así, se entiende que las evidencias de esta investigación sirven para auxiliar otras realidades para que puedan intervenir con la auriculoterapia, así favoreciendo la salud del trabajador. 

## Conclusión

Se constataron los beneficios de la auriculoterapia para el alivio de ansiedad y estrés de profesionales de enfermería perioperatoria. La auriculoterapia, aplicada con puntos verdaderos y *sham* (placebos), resultó con igual efecto para la ansiedad y el estrés de los profesionales investigados. Las evidencias fueron reforzadas con declaraciones que revelaron las contribuciones acerca del uso de esta práctica. Se constató la importancia de intervenciones no farmacológicas, como la auriculoterapia, con la finalidad de auxiliar la recuperación y promoción de la salud de los trabajadores de enfermería perioperatoria. 

Sugerimos que estudios futuros investiguen la eficacia de la auriculoterapia en otras realidades y con otras categorías profesionales. También, la comparación de la técnica con diferentes materiales, como agujas semipermanentes *versus* semillas o cristales, constituyen otras posibilidades de investigación.
